# Role of Organ-Specific Endothelial Cells in Melanoma Adhesion Patterns

**DOI:** 10.3390/biomedicines14071409

**Published:** 2026-06-23

**Authors:** Marwa Hamdan, István Szász, Tünde Várvölgyi, Margit Balázs, Viktória Koroknai

**Affiliations:** 1 Department of Public Health and Epidemiology, Faculty of Medicine, University of Debrecen, 4028 Debrecen, Hungary; hamdan.marwa@med.unideb.hu (M.H.); szasz.istvan@med.unideb.hu (I.S.); balazs.margit@med.unideb.hu (M.B.); 2 Doctoral School of Health Sciences, University of Debrecen, 4032 Debrecen, Hungary; 3 HUN-REN-UD Public Health Research Group, Department of Public Health and Epidemiology, Faculty of Medicine, University of Debrecen, 4032 Debrecen, Hungary; 4 Department of Dermatology, Faculty of Medicine, University of Debrecen, 4032 Debrecen, Hungary; varvolgyi.tunde@med.unideb.hu

**Keywords:** melanoma metastasis, cell adhesion, endothelial environment, extracellular matrix, integrins

## Abstract

**Background**: The metastatic dissemination of melanoma involves adhesion of circulating tumor cells within organ-specific vascular beds; however, the relative contribution of the endothelial environment versus that of the melanoma-intrinsic molecular state remains unclear. **Materials and Methods**: We quantified the in vitro adhesion of primary (*n* = 5) and metastatic (*n* = 3) melanoma cell lines to human hepatic, brain, and pulmonary endothelial cells under co-culture conditions, and we profiled the expression of 86 adhesion- and extracellular-matrix-related genes in melanoma and endothelial cells. **Results**: Adhesion was highest for the hepatic endothelium, intermediate for the pulmonary endothelium, and lowest for the brain endothelium. This endothelial preference was conserved in both primary and metastatic melanoma cells, though metastatic cells exhibited higher absolute adhesion. The linear mixed-effect models revealed that the effects of adhesion state on melanoma gene expression were modest and varied by endothelial type, whereas melanoma origin had more widespread and larger effects (mean absolute standardized coefficients of 0.32–0.47 versus 0.60–0.87, respectively). The expression of three genes (*SPP1*, *ITGA11*, and *MMP2*) was associated with melanoma origin in all endothelial types. Spearman’s co-expression analysis revealed endothelial-type-specific gene networks, and within-sample permutation confirmed the non-random coordination in all three endothelial types. **Conclusions**: Our findings support a model in which endothelial organ specificity contributes to melanoma cell adhesion behavior and associated transcriptional patterns, highlighting the importance of the vascular interface as a biologically active mediator of early metastatic cell–endothelium interactions.

## 1. Introduction

Cutaneous malignant melanoma is among the most aggressive forms of skin cancer and accounts for the majority of skin cancer-related deaths, driven by its pronounced propensity for early metastatic dissemination to visceral organs such as the liver, lung, and brain [1,2,3,4,5,6]. Clinical outcomes differ substantially across metastatic sites, reflecting site-specific biological constraints on progression [7]. To establish secondary lesions, circulating melanoma cells must arrest in the vasculature, adhere to endothelial surfaces, and transmigrate into the target organ [7,8,9,10,11,12]. The endothelium is an active regulator that governs adhesion, signaling, permeability, and immune trafficking, thereby shaping and limiting colonization [13,14].

Identical oncogenic drivers occur in melanomas with divergent metastatic trajectories and organ preferences [6], showing that genotype alone does not explain metastatic efficiency or organ selectivity. Metastatic success depends on compatibility between disseminated cells and the microenvironments encountered at secondary sites [15,16].

This compatibility is mediated, in part, by alterations in adhesion-related proteins, including integrins, transmembrane receptors that link the extracellular matrix (ECM) to the actin cytoskeleton [6,17,18,19]. Integrin complexes and their unique ECM ligands (such as laminins and collagens) control the strength and organ specificity of tumor cell anchoring, whereas matrix remodeling enzymes (such as matrix metalloproteinases (MMPs)) allow for localized microenvironmental adaptations [20,21].

Specific integrins, including αvβ3, α5β1, α4β1, and β1-containing receptors, have been implicated in tumor adhesion, vascular arrest, and progression [9,22,23], yet integrin expression alone does not predict function because receptor activity depends on associated molecular complexes and endothelial-defined ligand environments [19,24]. Additional adhesion systems, including immunoglobulin superfamily members such as ICAM-1, VCAM-1, PECAM-1, selectins, and MCAM/CD146, also regulate rolling, firm adhesion, junctional remodeling, and transendothelial migration in an endothelial-dependent manner [13,25,26,27,28]. Adhesion, therefore, reflects interactions between heterogeneous melanoma states and heterogeneous endothelial environments.

The vascular endothelium represents a critical determinant of microenvironmental compatibility, displaying substantial heterogeneity across organs with respect to transcriptional profiles, surface receptor composition, ECM organization, and responses to mechanical and inflammatory cues [13,15,25,29,30]. Notably, liver sinusoidal, pulmonary microvascular, and brain microvascular endothelia differ in fenestration, junctional architecture, basement-membrane composition, and immune surveillance, establishing different vascular niches that shape tumor–endothelium interactions [24,25,31]. A central limitation in addressing this complexity is that melanoma experimental studies often rely on reductionist models that treat the endothelium as a uniform entity, with adhesion examined using a single endothelial type under the assumption of generalizability across vascular beds [32]. A knowledge gap therefore remains: the extent to which the transcriptional networks governing melanoma adhesion are intrinsic to the tumor metastatic state versus extrinsically organized by the endothelial microenvironment of the target organ is unknown. In this study, we investigated the in vitro adhesion capacity and transcriptomic responses of primary and metastatic melanoma cells after co-culturing with three distinct types of human endothelial cells (hepatic, pulmonary, and brain), which represent clinically relevant sites of melanoma metastasis. Through targeted expression profiling of adhesion- and ECM-related genes, along with mixed-effect statistical modeling, we aimed to understand how endothelial organ specificity associates with molecular networks governing early metastatic cell–endothelium interactions.

## 2. Materials and Methods

### 2.1. Melanoma Cell Lines and Cell Cultures

A panel of 15 human melanoma cell lines derived from primary tumors and metastatic lesions was initially analyzed. The primary melanoma cell lines (*n* = 11) comprised WM35, WM3211, WM1361, WM1366, WM278, WM793B, WM983A, WM115, A375, WM3248, and Mel1617, and the metastasis-derived cell lines (*n* = 4) consisted of WM983B, A2058, M24, and WM266-4. The clinicopathological characteristics of the melanoma cell lines are summarized in Supplementary Table S1. The melanoma cell lines were obtained from different sources: 5 cell lines (WM35, A375, Mel1617, A2058, and M24) were purchased from the American Type Culture Collection (ATCC, Manassas, VA, USA); 8 cell lines (WM793B, WM3211, WM1361, WM278, WM983A, WM1366, WM3248, and WM983B) were obtained from the Coriell Institute for Medical Research (Camden, NJ, USA); and 2 cell lines (WM115 and WM266-4) were obtained from Rockland Immunochemicals, Inc. (Limerick, PA, USA).

The melanoma cell lines were cultured in RPMI 1640 medium supplemented with stable glutamine (Biowest, Nuaillé, France) and 10% fetal bovine serum (FBS; Gibco, Carlsbad, CA, USA) at 37 °C in a humidified incubator with 5% CO_2_.

### 2.2. Endothelial Cell Cultures

Primary human hepatic sinusoidal endothelial cells (HHSECs), brain microvascular endothelial cells (HBMECs), and pulmonary microvascular endothelial cells (HPMECs) were obtained from ScienCell Research Laboratories (Carlsbad, CA, USA). Cells were cultured according to the manufacturers’ protocols in fibronectin-coated culture vessels (2 µg/cm^2^) using Endothelial Cell Medium (ECM, ScienCell) supplemented with 1% endothelial cell growth supplement (ECGS; #1052), 1% penicillin/streptomycin (P/S; #0503), and 5% FBS (#0025). Endothelial cells were maintained at 37 °C in a humidified incubator with 5% CO_2_ and used within the recommended passage range.

### 2.3. Melanoma Tissue Samples

Melanoma tissue samples were obtained from the Dermatology Clinic of the University of Debrecen Clinical Centre, University of Debrecen (Debrecen, Hungary); these samples were derived from patients who had not received any treatment prior to the surgical excision of their primary lesions. Tumors were diagnosed based on formalin-fixed, paraffin-embedded (FFPE) tissue sections stained with hematoxylin and eosin. A total of 20 tumor samples were examined using qRT-PCR (LightCycler 480 Instrument II, Roche Diagnostics, GmbH, Mannheim, Germany). The clinical–pathological parameters of the tumor samples are summarized in Supplementary Table S2. This study was approved by the Ethics Committee of the Hungarian Scientific Council for Health [document numbers: TUKEB 17876–2018/EKU and BMEU/715-1/2022/EKU] and was performed according to the relevant guidelines.

### 2.4. Fluorescent Labeling of Melanoma Cells

For adhesion assays, melanoma cells were labeled using the PKH26 Red Fluorescent Cell Linker Kit (Sigma-Aldrich, St. Louis, MO, USA) according to the manufacturer’s instructions. Cells were washed in a serum-free medium, resuspended in 1 mL of dilution buffer, and incubated with PKH26 dye (final concentration 4 × 10^−6^ M) for 5 min at room temperature. Labeling was quenched with an equal volume of FBS for 1 min, followed by three washes with complete medium to remove unbound dye.

### 2.5. In Vitro Quantitative Adhesion Assay

Melanoma cell adhesion to organ-specific endothelial cells was assessed using 8-well Lab-Tek II Chamber Slides (CC2 Glass Slide; Thermo Fisher Scientific, Waltham, MA, USA). The chambers were coated with fibronectin (10 µg/mL; Gibco, Carlsbad, CA, USA) prior to seeding HHSECs, HBMECs, or HPMECs, which were grown to full confluence. PKH26-labeled melanoma cells were seeded onto endothelial monolayers at 5 × 10^4^ cells per well and incubated for 15 min at room temperature under constant orbital agitation (Stuart SSM1 mini orbital shaker, Bibby Scientific, Stone, UK; 145 rpm) to introduce limited fluid movement. The 15 min incubation time was selected to capture initial specific tethering events before non-specific attachment and spreading, which mask organ-specific differences in adhesion. Non-adherent cells were removed, and monolayers were washed twice with pre-warmed PBS (Gibco). Adherent cells were quantified using a Zeiss LSM 700 confocal microscope (Carl Zeiss, Jena, Germany). Cells were counted in at least five representative fields per well, and adhesion was expressed as mean cell counts per field.

### 2.6. Separation of Adherent and Non-Adherent Melanoma Cells

To separate adherent and non-adherent melanoma cells for downstream expression analysis, adhesion assays were repeated for the primary cell lines (WM3211, WM793B) and metastatic derived cell lines (M24, WM983B) using 6-well plates coated with fibronectin (10 µg/mL). Melanoma cells were seeded at 2 × 10^5^ cells per well onto confluent endothelial monolayers. After incubation, non-adherent cells were gently collected, and the remaining adherent melanoma cells were washed twice with prewarmed PBS. To detach adherent melanoma cells without disrupting the endothelial monolayer, the wells were treated with 0.5 mM EDTA (Sigma-Aldrich, St. Louis, MO, USA) for 10 min at 37 °C, and adherent cells were collected and processed for RNA isolation.

### 2.7. RNA Isolation and Quantitative Real-Time PCR

Total RNA was isolated from melanoma cell lines and endothelial cells using a silica-membrane spin column-based kit (NucleoSpin RNA Kit; Macherey–Nagel, Düren, Germany). RNA concentration and purity were assessed using a NanoDrop ND-1000 spectrophotometer (NanoDrop Technologies, Wilmington, DE, USA), and only samples with an A260/A280 ratio ≥ 1.8 were used for downstream analyses.

Complementary DNA (cDNA) was synthesized from 1 µg total RNA using the High-Capacity cDNA Reverse Transcription Kit (Applied Biosystems Inc., Foster City, CA, USA). The expression of 86 integrin pathway-related genes and 8 housekeeping genes were quantified via real-time PCR using Xceed SyGreen qPCR Probe 2× Mix Hi- ROX (Institute of Applied Biotechnologies, Praha, Czech Republic) on a LightCycler 480 Instrument II (Roche Diagnostics, GmbH, Mannheim, Germany). The primers were obtained from the Human Integrin Signaling Primer Library (HINT-I; RealTimePrimers.com). Relative gene expression was calculated using the 2^−ΔCt^ method and is reported as mean values from two independent experiments.

### 2.8. Statistical Analysis

All quantitative analyses were performed in Python (v3.11, Python Software Foundation, Wilmington, DE, USA). Phenotypic adhesion differences were assessed using one-way ANOVA followed by Tukey’s HSD post hoc test, with statistical significance defined at *p* < 0.05. Gene expression values were log_2_-transformed prior to analysis. Missing values were imputed using the distance-weighted k-nearest neighbors algorithm (k = 3). Differences in gene expression levels associated with adhesion state, melanoma origin, and endothelial type were evaluated using linear mixed-effect models, including melanoma cell line as a random intercept, with the parameters estimated via restricted maximum likelihood (REML). To assess endothelium-dependent effects, models were stratified by endothelial type, melanoma origin, or adhesion state. In addition, marginal models were fitted to the pooled dataset, including endothelial origin as a fixed-effect covariate. *p*-values were adjusted for multiple comparisons using the two-stage adaptive Benjamini–Hochberg False Discovery Rate (FDR) method, yielding *q*-values [33], with a significance threshold of *q* < 0.10 (where *q* represents the FDR-adjusted *p*-value). Gene co-expression patterns were quantified using pairwise Spearman’s rank correlations within each endothelial type, with statistical calibration based on within-sample permutation (B = 200). Effect sizes are summarized using Hedges’ g and standardized regression coefficients. All statistical analyses were performed in Python (v3.11) using standard scientific libraries (i.e., pandas, NumPy, statsmodels, and SciPy). The complete statistical pipeline, model specifications, and all equations are provided in Supplementary Method S1.

## 3. Results

### 3.1. Gene Expression Patterns of Adhesion- and ECM-Related Genes in Melanoma Cell Lines, Melanoma Tumor Samples, and Endothelial Cells

**Figure 1:**
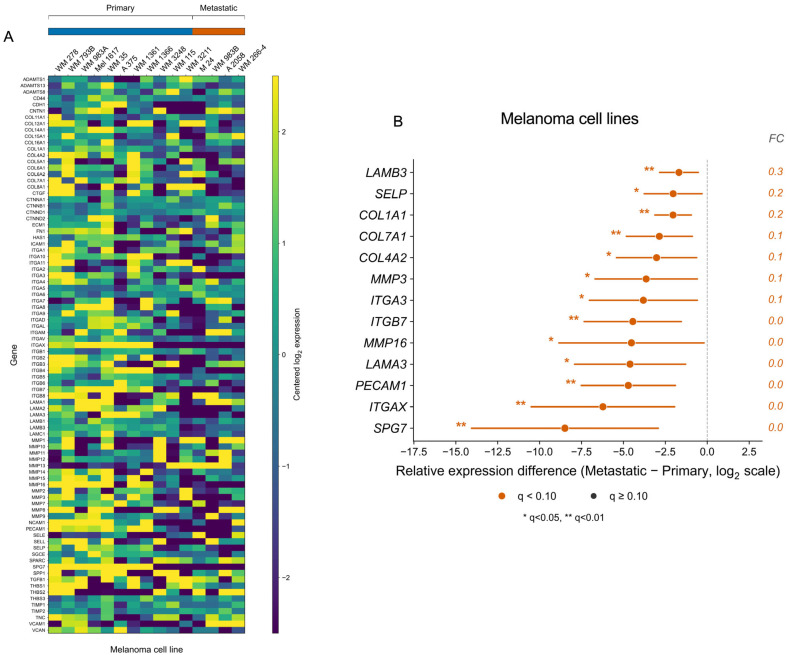
Gene expression profiles of melanoma cell lines. (**A**) Heatmap of log_2_-transformed gene expression values of 15 melanoma cell lines (*n* = 15). Columns represent melanoma cell lines grouped by primary or metastatic status; rows represent genes clustered by expression similarity. The results are shown as the means of two replicates. (**B**) Forest plot showing gene expression differences between metastatic and primary melanoma cell lines (*n* = 15). Points represent REML-fitted fixed-effect coefficients with 95% Wald CIs. Orange points indicate *q* < 0.1 (where *q* represents the False Discovery Rate (FDR)-adjusted *p*-value), with * *q* < 0.05 and ** *q* < 0.01.

Adhesion- and ECM-related gene expression patterns were first examined in a panel of primary- (*n* = 11) and metastasis-derived (*n* = 4) melanoma cell lines. Unsupervised hierarchical clustering of log_2_-transformed data revealed substantial heterogeneity in the expression of adhesion- and ECM-related genes across melanoma cell lines (Figure 1A). However, differential expression analysis using per-gene linear mixed-effect models, with cell line as a random intercept, identified 13 genes that significantly distinguished primary from metastatic cell lines (*q* < 0.05), namely, *LAMB3*, *SELP*, *COL1A1*, *COL7A1*, *COL4A2*, *MMP3*, *ITGA3*, *ITGB7*, *MMP16*, *LAMA3*, *PECAM1*, *ITGAX*, and *SPG7* (Figure 1B).

**Figure 2:**
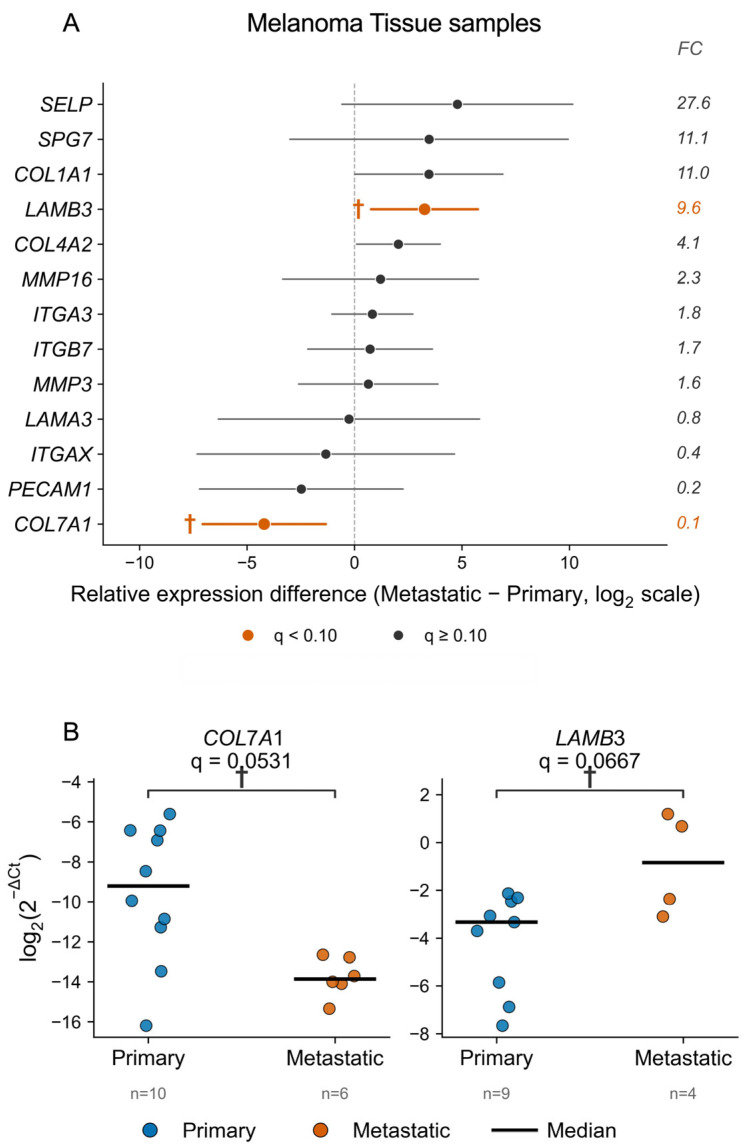
Gene expression differences in melanoma tissue samples. (**A**) Forest plot showing gene expression differences between metastatic and primary melanoma tissue samples (*n* = 20). Points represent REML-fitted fixed-effect coefficients with 95% Wald CIs. Orange points indicate *q* < 0.1 (where *q* represents FDR-adjusted *p*-value), and black points indicate not significant (*q* > 0.10). † *q* < 0.1. (**B**) Strip plots of expression values of *COL7A1* and *LAMB3*, the two genes meeting the FDR threshold of *q* ≤ 0.10, in individual tissue samples. Horizontal black lines denote group medians. Blue: primary; orange: metastatic. Per-group sample sizes are annotated below each strip. † *q* < 0.1.

To evaluate whether these cell-line observations extend to clinical samples, the expression differences in the 13 identified genes were quantified in an independent cohort of primary and metastatic melanoma tissue samples (*n* = 20) using per-gene linear mixed-effect models with sample as a random intercept. Of the genes tested, two genes—*COL7A1* (*q* = 0.053) and *LAMB3* (*q* = 0.067)—showed significant differences. Specifically, *COL7A1* had higher expression in the primary tissue samples, whereas *LAMB3* was more highly expressed in the metastatic tissue samples (Figure 2A,B). Despite considerable within-group variability, clear differences between the primary and metastatic tissue samples were observed for these two genes. The expression of the remaining genes did not reach a significant difference in the tissue samples. Interestingly, the cell line analysis showed higher expression of both genes in the primary cell lines, contrasting with the finding of tissue-level upregulation of *LAMB3* in metastatic tissue samples (Supplementary Figures S1 and S2).

**Figure 3:**
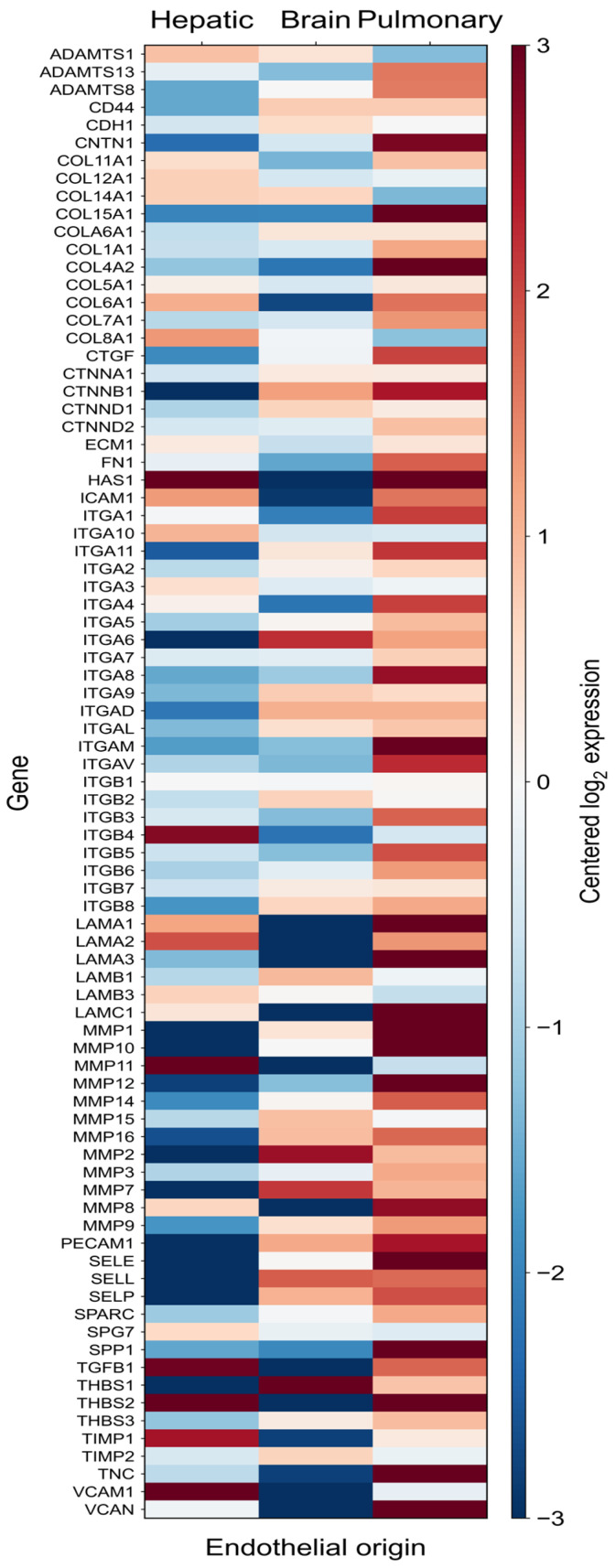
Gene expression profiles of hepatic, brain, and pulmonary endothelial cells. Heatmap of selected adhesion- and ECM-related genes showing row-wise, mean-centered expression in hepatic, brain, and pulmonary endothelial cells. The results are shown as the means of two replicates.

Adhesion- and ECM-related gene expression patterns were also profiled in the hepatic, brain, and pulmonary endothelial cells used in the adhesion assays (shown as the mean of two independent experiments; Figure 3). Each endothelial type exhibited a distinct expression profile of integrin subunits, junctional molecules, ECM components, and matrix-remodeling enzymes. Hepatic endothelial cells showed high expression of *HAS1*, *VCAM1*, *ITGB4*, and *TGFB1*; brain endothelial cells showed elevated expression of *ITGA6*, *MMP2*, *SELL*, *SELP*, and *THBS1*; and pulmonary endothelial cells exhibited elevated expression of *COL15A1*, *COL4A2*, *ICAM-1*, *SELE*, *SELP*, and several integrin alpha subunits (*ITGA8*, *ITGAV*, *ITGA4*).

### 3.2. Identification of Adhesion Molecular Patterns in Melanoma Cell Lines and Associated Gene Expression Profiles Across Brain, Hepatic, and Pulmonary Endothelial Types

**Figure 4:**
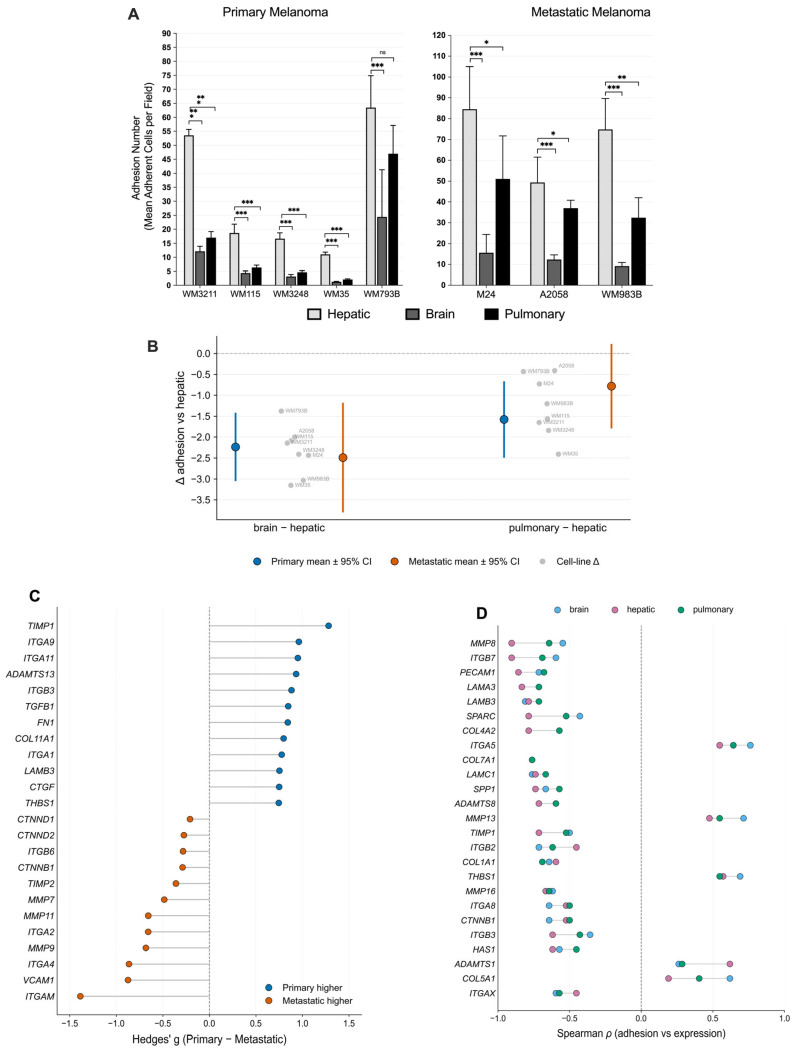
Melanoma adhesion patterns across endothelial types and associated gene expression profiles. (**A**) Adhesion of melanoma cells to brain, hepatic, and pulmonary endothelial monolayers. Left: primary melanoma cell lines (*n* = 5) and metastatic melanoma cell lines (*n* = 3) are shown on the left and right, respectively. Bars indicate the mean ± SD of number of adherent cells per microscopic field from three independent replicates per cell line. Statistical significance within each melanoma group was assessed using one-way ANOVA with Tukey’s HSD post hoc test. ns: not significant, * *p* < 0.05; ** *p* < 0.01; *** *p* < 0.001. (**B**) Cell line-specific adhesion difference between endothelial cell types (brain vs. hepatic and pulmonary vs. hepatic). Points represent individual cell-line contrasts; black and blue markers denote group means ±95% CIs for primary and metastatic melanoma, respectively. (**C**) Forest plot of standardized gene expression differences (Hedges’ g; primary vs. metastatic) for adhesion- and ECM-related genes with the largest absolute effect sizes. (**D**) Spearman’s rank correlations between melanoma gene expression and organ-specific endothelial adhesion. Each point represents a single gene–endothelium correlation; colors indicate endothelial origin.

To ensure a representative biological diversity for downstream functional and molecular analyses, eight melanoma cell lines (primary, *n* = 5; metastatic, *n* = 3) were selected for functional adhesion assays based on the hierarchical clustering patterns observed in the initial screening (Figure 1A). Adhesion to the hepatic, pulmonary, and brain endothelial monolayers is quantified as the mean number of adherent melanoma cells per microscopic field (mean of three independent experiments per cell line per endothelial type). Within both the primary and metastatic melanoma groups of cell lines, one-way ANOVA with Tukey’s HSD post hoc correction revealed significant differences in adhesion across endothelial types (Figure 4A). Adhesion was highest for the hepatic endothelium, intermediate for the pulmonary endothelium, and lowest for the brain endothelium. This organ-specific hierarchy was consistently observed in both primary and metastatic melanoma cell lines, although absolute adhesion values were elevated in metastatic-derived cell lines. Representative fluorescence micrographs illustrate this pattern (Supplementary Figure S3).

To assess endothelial preference independently of baseline adhesion capacity, within-cell-line contrasts were computed relative to hepatic endothelium, as it consistently showed the highest levels of adhesion in our assays (Figure 4B). Both primary and metastatic melanoma cell lines showed strongly negative (brain–hepatic) contrasts and more modest (pulmonary–hepatic) contrasts. Group-level analyses revealed conserved directionality across melanoma cell lines. Notably, metastasis-derived melanoma cells showed the adhesion pattern observed in primary-tumor-originated cells while exhibiting higher absolute adhesion.

Gene expression differences between primary and metastatic melanoma cell lines were quantified using Hedges’ g to measure effect sizes. The results showed that the distinction between the two groups of cell lines was driven by a collective shift across multiple adhesion- and ECM-related genes, rather than a massive change in any single dominant gene (Figure 4C). The largest effect sizes representing higher expression (upregulation) in primary melanoma were observed in *TIMP1*, *ITGA9*, *ITGA11*, and *ADAMTS13*, whereas those indicating upregulation in metastatic melanoma were associated with *VCAM1*, *ITGA4*, *ITGA2*, and *MMP9*.

The relationship between gene expression and adhesion was evaluated using Spearman’s rank correlations within each endothelial type (Figure 4D). Adhesion to the brain endothelium was positively correlated with expression of *COL5A1*, *MMP13*, and *THBS1*, whereas adhesion to the hepatic endothelium was negatively correlated with expression of *SPARC*, *COL4A2*, *TIMP1*, and *ITGB3*. Expression of *COL7A1* and *ITGB2* was negatively correlated with adhesion to the pulmonary endothelium. Expression of *ITGA5* and *THBS1* was positively correlated with adhesion across all endothelial types. Conversely, *MMP8*, *ITGB7*, *SPP1*, *COL1A1*, *PECAM1*, and *LAMB3* showed a negative correlation with adhesion in all endothelial types.

### 3.3. Characterizing the Endothelial-Dependent Gene Expression Shifts in Primary and Metastatic Melanoma Cells

To assess gene expression changes during adhesion, melanoma cells were separated after co-culture into non-adherent (supernatant) and adherent (endothelium-bound) fractions. Gene expression was compared using gene-wise linear mixed-effect models fitted within each endothelial type (brain, hepatic, and pulmonary). The effects of adhesion state (adherent minus non-adherent) were estimated while adjusting for melanoma origin, with cell line included as a random intercept.

**Figure 5:**
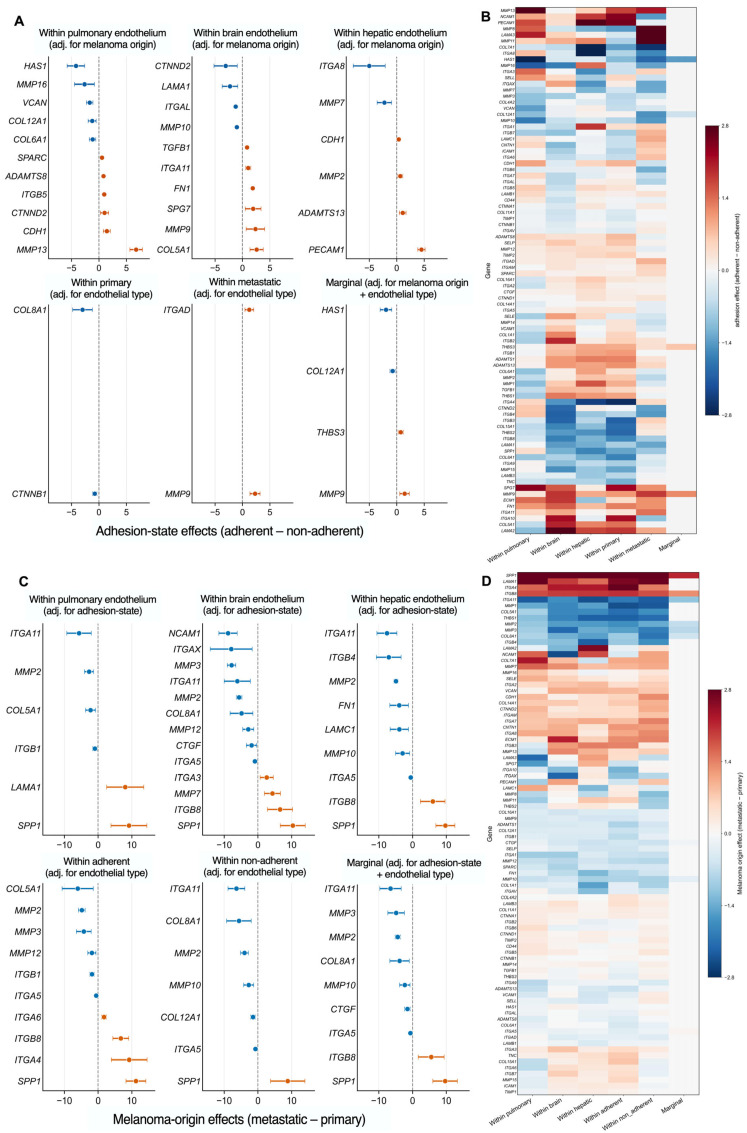
Effects of adhesion state on melanoma gene expression. Forest plots of adhesion-state effects: (**A**) (adherent minus non-adherent) and melanoma-origin effects; (**B**) (metastatic minus primary) on log_2_ gene expression. In each panel, the top row shows estimates within each endothelial type, adjusted for the complementary covariate. The bottom row shows estimates within each stratum of the complementary covariate, adjusted for endothelial type (left, center), and marginal estimates adjusted for both covariates (right). Points represent fixed-effect beta coefficients; horizontal bars indicate 95% Wald coefficients intervals. Only genes with *q* < 0.10 (two-stage BH, applied per stratum) are shown; null-result sub-panels are annotated. Heatmaps of z-scored fixed-effect estimates for adhesion-state (**C**) and melanoma-origin (**D**) effects. Columns correspond to endothelial-specific strata and the marginal model; rows represent genes ordered by average-linkage clustering. Red indicates higher expression in adherent (**C**) or metastatic (**D**) cells; blue indicates higher expression in non-adherent (**C**) or primary (**D**) cells. All models were fitted using REML, with cell line as a random intercept.

The adhesion-responsive gene sets differed by endothelial type (Figure 5A, Table 1). The highest number of significant genes was observed in melanoma cells within the pulmonary endothelium (11 of 86; *q* < 0.10), with decreased expression of *HAS1*, *MMP16*, *VCAN*, *COL12A1*, and *COL6A1* and increased expression of *MMP13*, *CDH1*, *ADAMTS8*, *ITGB5*, *CTNND2*, and *SPARC* in adherent cells compared to non-adherent cells. Analysis of melanoma cells within the hepatic endothelium yielded a six-gene set with decreased expression of *ITGA8* and *MMP7* and increased expression of *MMP2*, *ADAMTS13*, *CDH1*, and *PECAM1*, sharing only *CDH1* with the pulmonary set. Analysis of melanoma cells within the brain endothelium showed ten significant genes, including increased expression of *COL5A1*, *MMP9*, *SPG7*, *ITGA11*, *FN1*, and *TGFB1* and decreased expression of *CTNND1*, *LAMA1*, *ITGAL*, and *MMP10*. Stratification by melanoma origin identified two significant genes (*COL8A1*, *CTNNB1*) in the primary melanoma group, and two genes (*ITGAD*, *MMP9*) in the metastatic melanoma group (Figure 5A, Table 2).

We also tested for general adhesion signature by pooling all endothelial types together into a single model (the marginal model). The marginal adhesion-state models identified four genes (*HAS1*, *COL12A1*, *THBS3,* and *MMP9*) with lower mean absolute standardized coefficients than those observed in any endothelial type-specific analysis (0.32 versus 0.37–0.47; Table 1).

In parallel with the estimation of adhesion-state effects, we estimated melanoma-origin effects (metastatic minus primary) using gene-wise linear mixed-effect models fitted within each endothelial type, adjusting for adhesion state. Melanoma-origin-associated expression differences were more widespread and substantially larger than adhesion-state effects across all subgroups (Table 1). Analysis of the brain endothelium yielded 13 significant genes, the highest number among all comparisons, comprising *NCAM1*, *ITGAX*, *MMP3*, *ITGA11*, *MMP2*, *COL8A1*, *MMP12*, *CTGF*, *ITGA5*, *ITGA3*, *MMP7*, *ITGB8*, and *SPP1*. Analysis of the hepatic endothelium revealed nine significant genes (*ITGA11*, *ITGB4*, *MMP2*, *FN1*, *LAMC1*, *MMP10*, *ITGA5*, *ITGB8*, and *SPP1*), while analysis of the pulmonary endothelium uncovered six (*ITGA11*, *MMP2*, *COL5A1*, *ITGB1*, *LAMA1*, *SPP1*). The expression of *SPP1*, *ITGA11*, and *MMP2* was significantly different across all endothelial types; *SPP1* was consistently upregulated in metastatic melanoma, whereas *ITGA11* and MMP2 showed higher expression in primary melanoma (Figure 5B).

Stratification by adhesion state revealed more origin-associated genes in adherent cells than in non-adherent cells (10 vs. 7 genes), namely, *COL5A1*, *MMP2*, *MMP3*, *MMP12*, *ITGB1*, *ITGA5*, *ITGA6*, *ITGB8*, *ITGA4*, and *SPP1* in adherent cells and *ITGA11*, *COL8A1*, *MMP2*, *MMP10*, *COL12A1*, *ITGA5*, and *SPP1* in non-adherent cells (Figure 5B). The marginal models identified nine genes, with *SPP1* and *ITGA11* showing the largest standardized effects (Figure 5B: marginal panel, Table 2).

Two features emerged from these analyses. First, the identity and direction of adhesion-responsive genes were strongly dependent on endothelial type, with minimal overlap with organs. Second, the adhesion-state effects were smaller in both number and magnitude compared with the melanoma origin-associated effects. Melanoma origin-associated differences in gene expression were widespread but not fixed, varying by endothelial type.

The heatmaps of the z-scored beta coefficients illustrate these patterns; the adhesion state-associated coefficients clustered by endothelial type and showed divergent inter-organ directionality (Figure 5C), whereas the origin-associated coefficients exhibited partial cross-organ concordance modulated by endothelial environment (Figure 5D). The full linear mixed-effect model results are available in Supplementary Tables S3–S6.

### 3.4. Divergent Gene Co-Expression Patterns in Melanoma Across Distinct Endothelial Types

**Figure 6:**
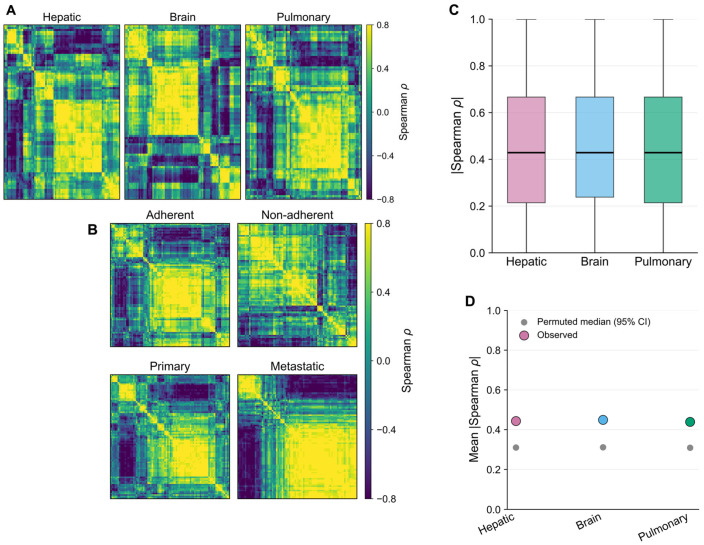
Gene co-expression patterns in melanoma across distinct endothelial types. (**A**) Heatmaps showing pairwise Spearman’s rank correlation (ρ) matrices computed separately for each endothelial type (hepatic, brain, and pulmonary). Genes are reordered by average-linkage hierarchical clustering. (**B**) Spearman’s ρ matrices stratified by adhesion state and melanoma origin, averaged element-wise across endothelial types within each stratum. (**C**) Distribution of pairwise |Spearman’s ρ| per endothelial type. Boxes indicate interquartile range, center lines denote medians, and whiskers indicate the full data range. (**D**) Comparison of observed mean absolute Spearman’s correlation magnitudes within each endothelial type to null expectations derived from permutation testing. Colored points indicate observed mean |ρ| values, gray points indicate median |ρ| values from permuted datasets, and vertical lines denote the 2.5th–97.5th percentile range of the permutation distribution.

The mixed-effect models quantified the mean expression differences but did not capture how gene expression patterns change across melanoma cell lines within a defined endothelial environment. Therefore, gene co-expression patterns were examined using Spearman’s rank correlations computed across melanoma cell lines within each endothelial type. For each endothelial type, pairwise correlations were calculated based on the log_2_-transformed expression profiles of all genes, yielding endothelial-type-specific correlation matrices (Figure 6A).

The resulting correlation matrices revealed distinct networks of interactions in the hepatic, brain, and pulmonary endothelia. The block structure differed between endothelial types: gene pairs that were positively correlated in one endothelial type were either uncoupled or negatively correlated in another. For example, specific gene subsets showed strong positive correlations within the hepatic endothelium but were not correlated in the brain endothelium. However, correlations between genes were partially similar for the hepatic endothelium and pulmonary endothelium (Figure 6A). These findings indicate gene co-expression patterns are organized not only by an intrinsic melanoma program, but also by the endothelial type.

We also examined gene co-expression patterns across adhesion states (adherent versus non-adherent) and melanoma origins (primary versus metastatic) (Figure 6B). This analysis did not show the patterns observed when data were grouped by endothelial type. Adherent cells showed completely different gene co-expression patterns compared to non-adherent cells. However, metastatic melanoma showed subsets of positively correlated genes that were partially consistent with those observed in primary melanoma. The overall structure lacked a clear, organ-specific organization found in the analysis stratified by endothelial type. These results suggest that endothelial type exerts a stronger organizational influence on gene–gene interactions.

The strength of correlations, summarized as |Spearman’s ρ|, was moderate to strong (|ρ| > 0.6) in all three endothelial types, with roughly similar medians (Figure 6C). These findings suggest that biological variation is driven by the reorganization of gene networks rather than changes in correlation strength.

As a control test, permutation testing (*n* = 200 permutations) was performed for each endothelial type by shuffling the gene expression values independently within each sample, preserving per-sample marginal distributions while eliminating inter-gene dependence. In all three endothelial types, the observed mean |ρ| exceeded the 97.5th percentile of the permutation null distribution (Figure 6D), confirming that the observed correlation strength is non-random. This uniform pattern across all three endothelial types confirms that the observed patterns represent authentic biological phenomena rather than stochastic noise or organ-specific artifacts.

## 4. Discussion

Melanoma is among the most aggressive and treatment-resistant human cancers, with a rising global incidence [34]. Extensive efforts have characterized the genetic and transcriptional landscape of melanoma and its tumor microenvironment [1]. Cell adhesion is a central process in malignant transformation, metastatic dissemination, and therapy resistance [35]. However, the relative contributions of tumor-intrinsic factors and the endothelial environment to melanoma adhesion remain incompletely understood.

In the present study, we integrated quantitative adhesion assays, targeted gene expression profiling, and effect-focused statistical modeling across organ-specific endothelial types. Our findings demonstrate that endothelial type is strongly associated with melanoma cell adhesion behavior. Adhesion was observed to be highest for the hepatic endothelium, intermediate for the pulmonary endothelium, and lowest for the brain endothelium. This hierarchy was preserved in both primary and metastatic melanoma cells. Although metastatic-derived cells exhibited higher overall adhesion, the observed organ preference remained unchanged. These observations are consistent with known differences in endothelial structure, permeability, and extracellular matrix composition and organization across organs [13,25,30,36]. From a clinical perspective, the observed adhesion hierarchy exhibited only partial concordance with reported clinical metastatic frequencies. The relatively high adhesion to the hepatic endothelium is consistent with the liver being a frequent site (48%) of visceral melanoma metastasis, and the comparatively low adhesion to the brain endothelium may reflect the lower prevalence of brain metastasis (29%) [37,38]. However, although the lung is among the most common visceral sites of melanoma metastasis, we observed intermediate adhesion to the pulmonary endothelium. This discrepancy is not unexpected, as adhesion represents only the initial step of the metastatic cascade, and clinical organotropism additionally reflects extravasation, survival, and colonization; the comparison is therefore presented as partial concordance and not as direct correspondence.

These organ-specific adhesion patterns are further reflected at the transcriptional level. Despite the marked heterogeneity observed across melanoma cell lines, differential expression analysis identified 13 genes that significantly distinguished primary and metastatic melanomas [7,19]. Similar patterns have been reported regarding melanoma invasion and early dissemination [16,39].

Analysis of an independent cohort of melanoma tissue samples provided partial validation. Two genes, *COL7A1* and *LAMB3*, reached statistical significance (*q* ≤ 0.10) in mixed-effect modeling. The downregulation of *COL7A1* in metastatic tissue samples and cell lines is consistent with its proposed role in maintaining basement membrane integrity, and the loss of this gene has been associated with increased invasiveness [40]. In contrast, the opposite direction of *LAMB3* expression in tissues and cell lines may suggest modulation by the in vivo microenvironment [41,42]. This partial concordance between cell lines and tissues is consistent with the well-documented transcriptional divergence between cancer cell lines and primary tumors. The gene expression profiles derived from cell lines often cluster separately from clinical specimens, reflecting adaptation to prolonged in vitro culture and the absence of the native tumor microenvironment. In contrast, bulk tumor tissues comprise variable proportions of malignant, stromal, endothelial, and immune cells, which can dilute or mask melanoma-cell-intrinsic transcriptional signals. In addition, the in vivo microenvironment can also provide paracrine and mechanical factors that are not reproduced under standard culture conditions. These factors may explain why only a limited number of genes reached statistical significance in the tissue cohort and could also account for the discordant direction of LAMB3 expression between the cell line and tumor tissue datasets [43,44,45].

Studying the effects of adhesion state and melanoma origin (primary or metastatic) on gene expression within endothelial types provided a more detailed view of gene expression patterns. The identity and direction of adhesion-responsive genes varied by endothelial type, with minimal overlap across the pulmonary, hepatic, and brain endothelia. In contrast, melanoma-origin-associated expression effects were more widespread and of greater magnitude than adhesion-state effects under all the conditions tested.

A limited set of genes (*SPP1*, *ITGA11*, and *MMP2*) were significant across all three endothelial types. *SPP1* showed consistently higher expression in metastatic melanoma, aligning with the reported roles of its protein product, Osteopontin (OPN), in pro-survival signaling and invasive behavior through integrin-mediated adhesion [46]. Notably, these findings support previous functional studies from our group, which demonstrated that siRNA-mediated silencing of OPN significantly impairs melanoma cell proliferation and invasive capacity, a phenotype driven by the downstream modulation of key oncogenic regulators, including EGFR and tenascin C [47]. Furthermore, our group’s extensive characterization of OPN isoforms revealed that the relative expression of OPNa, OPNb and, especially, OPNc is significantly elevated in metastatic tumors compared to primary lesions. Specifically, OPNc expression correlates positively with increased Breslow thickness and the presence of metastasis, while its known receptor, integrin ITGB3, is selectively detectable in aggressive nodular subtypes [48,49,50].

*MMP7* expression was higher in metastatic melanoma cells within the brain endothelium, aligning with its reported association with poor prognosis [51]. *ITGB8* and *ITGA6*, which reached significance in multiple subgroups, have also been implicated in epithelial–mesenchymal transition and tumor progression, respectively [52,53]. These recurrent genes may represent components of a shared metastatic program that operates within, not independently of, endothelial-dependent expression programs.

The gene co-expression analyses extended these findings beyond mean expression differences. Spearman’s correlations varied across hepatic, pulmonary, and brain endothelial types and were not recovered by grouping based on melanoma origin or adhesion state alone. Permutation testing confirmed that the observed correlation strengths exceeded the null expectations in all three endothelial types. These results suggest that endothelial type may be associated not only with differences in adhesion and gene expression, but also with the network of gene interactions linking expression states to adhesive behavior. This multilevel organizational role is consistent with endothelial regulation of ligand presentation, junctional dynamics, and mechanotransductive signaling [15,29].

Endothelial gene expression profiling provided a potential molecular basis for these observations. Endothelial cells from different organs exhibited distinct adhesion- and ECM-related expression profiles, which is in line with known differences in junctional organization, ECM composition, and barrier properties [13,25,30]. Previous studies have shown that modulation of the endothelial state can impose strong constraints on leukocyte and tumor cell adhesion independently of changes in the interacting cell type, supporting the role of endothelial state as an active determinant of adhesion permissiveness [14,27,54,55].

These results suggest a potentially endothelial-dependent nature of integrin function in metastasis. Integrins mediate adhesion and mechanotransduction by linking the extracellular matrix to the cytoskeleton [17,18], but their function is likely influenced by endothelial-defined ligand availability, receptor organization, and mechanical cues. Endothelial-type-dependent integrin behavior has been documented in multiple tumor types [24,56,57], and the limited clinical efficacy of integrin-targeted therapies may reflect this conditionality instead of redundancy [19,58,59].

Several limitations must be considered. The findings are associative and derived from targeted transcriptomic profiling; functional confirmation via gene silencing (siRNA or CRISPR/Cas9), overexpression, or integrin-blocking antibodies is beyond the scope of this study and is crucial in future research to determine the roles of *SPP1*, *ITGA11*, and *MMP2* in organ-specific adhesion. In addition, protein-level measurements were not performed; therefore, the concordance between transcript and protein expression remains to be established. Moreover, while the targeted integrin/ECM-focused expression panel captures determinants of firm adhesion and cytoskeletal anchoring within tumor cells, it does not encompass early tethering and rolling mechanisms mediated by carbohydrate-based ligands or the endothelial glycocalyx [19,60,61,62]. Although the use of primary human endothelial cells improves physiological relevance, in vitro culture may attenuate tissue-specific cues such as perivascular signaling, specialized basement membrane composition, and cytokine gradients [25,63,64,65]. In addition, our assay used orbital agitation to provide a controlled and uniform approximation of shear stress across all endothelial models. However, this approach cannot fully recapitulate organ-specific hemodynamic environments that influence adhesion and transmigration in vivo, where circulating melanoma cells experience distinct mechanical forces ranging from the low-shear hepatic sinusoids to the higher-shear pulmonary and cerebral microvasculature [66,67]. These physiological mechanical forces may modulate integrin activation and adhesion-molecule presentation, potentially altering the hierarchy of endothelial interactions observed in the present study. Therefore, microfluidic and in vivo models will be required to determine how physiological flow conditions shape endothelial selectivity and melanoma-cell recruitment [15,68,69]. The tissue validation cohort (*n* = 20) provided partial corroboration but was powered to detect only large effects, and a larger, clinically annotated cohort would be needed to resolve subtler expression differences at the tissue level. Finally, the use of established cell lines enables controlled comparisons but cannot capture the full spectrum of transcriptional plasticity present in patient-derived melanoma [1,42].

## 5. Conclusions

This study suggests that melanoma adhesion and its underlying transcriptome networks are significantly modulated by endothelial identity. While metastatic progression appears to increase overall adhesive capacity, it does not substantially alter the endothelial preference hierarchy. Furthermore, transcriptional responses, including those of candidate genes like *SPP1*, *ITGA11*, and *MMP2*, and gene co-expression networks are notably modulated by endothelial type as well rather than by melanoma origin only. Together, these findings highlight the potential importance of incorporating endothelial organ specificity into future experimental models of metastasis, biomarker discovery, and targeted therapeutic evaluations.

## Figures and Tables

**Table 1:** Signal density and magnitude of gene expression changes by adhesion state and melanoma origin.

Variable	Model	Contrast	Response Rate ^1^	Mean Absolute Effect Size ^2^
adhesion	context	adhesion | organ = brain	0.11	0.46
adhesion	context	adhesion | organ = hepatic	0.07	0.44
adhesion	context	adhesion | organ = pulmonary	0.13	0.37
adhesion	context	adhesion | origin = metastatic	0.02	0.37
adhesion	context	adhesion | origin = primary	0.02	0.47
adhesion	marginal	adhesion | marginal	0.05	0.32
origin	context	origin | adhesion = adherent	0.12	0.80
origin	context	origin | adhesion = non-adherent	0.08	0.67
origin	context	origin | organ = brain	0.15	0.87
origin	context	origin | organ = hepatic	0.10	0.79
origin	context	origin | organ = pulmonary	0.07	0.60
origin	marginal	origin | marginal	0.10	0.63

^1^ Response rate: proportion of the 86 assayed genes with stratum-specific FDR-adjusted *q* < 0.10 (two-stage Benjamini–Hochberg method). A response rate of 0.00 denotes that no gene reached significance within that condition after FDR correction. ^2^ Mean absolute effect size: average magnitude of standardized regression coefficients (beta coefficient/gene-wise SD) computed across all 86 assayed genes. Context-specific contrasts reflect models stratified by endothelial type, melanoma origin, or adhesion state; marginal contrasts estimate effects averaged across endothelial types via fixed-effect adjustment.

**Table 2:** Top-ranking gene expression changes by standardized magnitude for adhesion state and melanoma origin.

Model ^1^	Contrast	Gene	Estimate ^2^	−log10(*q* Value) ^3^	Effect Size ^4^
marginal	adhesion | marginal	*HAS1*	−1.95	1.63	−1.13
marginal	adhesion | marginal	*THBS3*	0.74	1.31	0.89
marginal	adhesion | marginal	*MMP9*	1.44	1.31	0.80
context	adhesion | organ = brain	*MMP9*	2.37	1.28	1.33
context	adhesion | organ = brain	*TGFB1*	0.83	7.25	1.12
context	adhesion | organ = brain	*CTTND2*	−3.06	1.33	−1.09
context	adhesion | organ = hepatic	*PECAM1*	4.52	37.98	1.59
context	adhesion | organ = hepatic	*ITGA8*	−5.00	1.87	−1.23
context	adhesion | organ = hepatic	*MMP7*	−2.24	1.87	−1.00
context	adhesion | organ = pulmonary	*HAS1*	−4.18	5.36	−2.42
context	adhesion | organ = pulmonary	*MMP13*	6.78	28.85	2.15
context	adhesion | organ = pulmonary	*MMP16*	−2.63	1.48	−1.19
context	adhesion | origin = metastatic	*ITGAD*	1.24	1.03	1.22
context	adhesion | origin = primary	*COL8A1*	−2.99	1.33	−0.97
context	adhesion | origin = primary	*CTNNB1*	−0.77	1.56	−0.78
marginal	origin | marginal	*MMP2*	−4.40	26.90	−1.82
marginal	origin | marginal	*ITGA11*	−6.54	2.96	−1.78
marginal	origin | marginal	*SPP1*	9.66	5.23	1.72
context	origin | organ = brain	*MMP3*	−7.80	32.50	−2.09
context	origin | organ = brain	*ITGAX*	−7.90	1.09	−1.99
context	origin | organ = brain	*MMP7*	4.31	2.28	1.91
context	origin | organ = hepatic	*ITGB4*	−7.06	2.49	−1.62
context	origin | organ = hepatic	*LAMC1*	−3.91	1.33	−1.61
context	origin | organ = hepatic	*FN1*	−3.97	1.33	−1.49
context	origin | organ = pulmonary	*LAMA1*	8.03	1.15	1.52
context	origin | adhesion = adherent	*ITGB1*	−1.81	5.40	−1.74
context	origin | adhesion = adherent	*ITGA4*	9.22	2.21	1.72
context	origin | adhesion = adherent	*COL5A1*	−6.03	1.09	−1.59
context	origin | adhesion = non-adherent	*COL12A1*	−1.44	2.68	−1.16

^1^ Results are presented for two different modeling approaches: (1) the marginal rows show the general effect across all endothelial types together with the melanoma origin (primary and metastatic) or adhesion state, while (2) the context rows show the effect within a specific endothelial type together with melanoma origin or adhesion state only. ^2^ Each estimate denotes the fixed-effect beta coefficient from the linear mixed-effect model (log_2_ scale). ^3^ Significance is denoted by −log10 transformed *q*-values (FDR-adjusted *p*-values). ^4^ Effect size is the standardized regression coefficient, defined as the beta coefficient divided by the gene-wise standard deviation of expression values.

## Data Availability

The data presented in this study are available from the corresponding author upon reasonable request.

## Supplementary Materials

The following supporting information can be downloaded at: https://www.mdpi.com/article/10.3390/biomedicines14071409/s1, Method S1: Statistical Analysis Pipeline; Figure S1: Gene expression differences between metastatic and primary melanoma cell lines; Figure S2: Gene expression differences between metastatic and primary melanoma tissue samples; Figure S3: Representative fluorescence microscopic images of adherent melanoma cells; Table S1: Clinicopathological characteristics of human melanoma cell lines; Table S2: Clinical–pathological parameters of the melanoma tumor samples; Table S3: Marginal model gene-level expression effects for adhesion-state adjusted for melanoma origin and endothelial type; Table S4: Marginal model results of gene-level expression effects for melanoma origin adjusted for adhesion state and endothelial type; Table S5: Results for all 86 tested genes of linear mixed-effect models fitted within each endothelial context to estimate adhesion-state effects on expression, adjusting for melanoma origin; Table S6: Results for all 86 tested genes of linear mixed-effect models fitted within each endothelial context to estimate melanoma-origin effects on expression, adjusting for adhesion-state.

## Abbreviations

The following abbreviations are used in this manuscript:

ECGS	Endothelial cell growth supplement
ECM	Extracellular matrix/Endothelial cell medium
EDTA	Ethylenediaminetetraacetic acid
FBS	Fetal bovine serum
FDR	False discovery rate
FFPE	Formalin-fixed paraffin-embedded
HINT-I	Human Integrin Signaling Primer Library
ICAM-1	Intercellular adhesion molecule 1
LMM	Linear mixed-effect model
MAPK	Mitogen-activated protein kinase
MCAM/CD146	Melanoma cell adhesion molecule
PECAM-1	Platelet endothelial cell adhesion molecule 1
PI3K–AKT	Phosphoinositide 3-kinase—Protein kinase B
REML	Restricted maximum likelihood
VCAM-1	Vascular cell adhesion molecule 1
