# Supplementary material for: Role of Organ-Specific Endothelial Cells in Melanoma Adhesion Patterns
Source: Biomedicines. 2026 Jun 23;14(7):1409. doi: 10.3390/biomedicines14071409 (PMC13405823; doi:10.3390/biomedicines14071409)
Supplement: Supplementary file 1 [file biomedicines-14-01409-s001.zip › Revised Supplementary material-final 22 june.pdf]

biomedicines

MDPI

Supplementary material

Role of Organ-Specific Endothelial Cells in Melanoma Adhesion Patterns

Marwa Hamdan [1,2], István Szász [1,3], Tünde Várvölgyi [4], Margit Balázs [1,3] and Viktória Koroknai [1,*]

[1] Department of Public Health and Epidemiology, Faculty of Medicine, University of Debrecen, 4028 Debrecen, Hungary; hamdan.marwa@med.unideb.hu (M.H.) ; szasz.istvan@med.unideb.hu (I.S.) ; balazs.margit@med.unideb.hu (M.B.)
[2] Doctoral School of Health Sciences, University of Debrecen, 4032 Debrecen, Hungary
[3] HUN-REN-UD Public Health Research Group, Department of Public Health and Epidemiology, Faculty of Medicine, University of Debrecen, 4032 Debrecen, Hungary
[4] Department of Dermatology, Faculty of Medicine, University of Debrecen, 4032 Debrecen, Hungary, varvolgyi.tunde@med.unideb.hu
* Correspondence: koroknai.viktoria@med.unideb.hu ;Tel.: +3652/512764/77162

Table of Contents

1. **Supplementary Method S1**: Statistical Analysis Pipeline
2. **Supplementary Figure S1**: Gene expression differences between metastatic and primary melanoma cell lines
3. **Supplementary Figure S2**: Gene expression differences between metastatic and primary melanoma tissue samples
4. **Supplementary Figure S3:** Representative fluorescence microscopic images of adherent melanoma cells
5. **Supplementary Table S1**: Clinicopathological characteristics of human melanoma cell lines
6. **Supplementary Table S2**: Clinical-pathological parameters of melanoma tumour samples
7. **Supplementary Table S3**: Marginal model gene-level expression effects for adhesion-state adjusted for melanoma origin and endothelial type
8. **Supplementary Table S4**: Marginal model results of gene-level expression effects for melanoma-origin adjusted for adhesion-state and endothelial type
9. **Supplementary Table S5**: Results for all 86 tested genes of linear mixed-effects models fitted within each endothelial context to estimate adhesion-state effects on expression, adjusting for melanoma-origin
10. **Supplementary Table S6**: Results for all 86 tested genes of linear mixed-effects models fitted within each endothelial context to estimate melanoma-origin effects on expression, adjusting for adhesion-state

1. Supplementary Method S1: Statistical Analysis Pipeline

S1.1 Data pre-processing

Gene expression values were $\log_2$-transformed prior to all downstream analyses. For raw intensity or count data with zero or negative entries, a stabilizing shift was applied:

$$\tilde{x} = \log_2(x + s), \quad \text{where} \quad s = \max(0, -\min(x) + 1) \qquad (1)$$

ensuring strict positivity of the argument. Missing expression values were imputed by k-nearest neighbours ($k = 3$) with inverse-distance weighting (scikit-learn, KNNImputer). Genes with entirely missing profiles were excluded from imputation and retained as missing. Adhesion scores were averaged over replicate microscopic field counts within each experimental unit prior to modelling.

For heatmap visualization, imputed $\log_2$-expression matrices were row-centered (gene-wise mean subtraction) and clipped to $[-c, c]$ with $c = 2.5$ or $c = 3.0$ to attenuate the influence of extreme values. Columns were ordered by hierarchical clustering (average-linkage on Pearson correlation distance, computed within each melanoma-status stratum).

S1.2 Linear mixed-effects models

Gene-wise expression differences associated with adhesion state, melanoma origin, and endothelial context were estimated using linear mixed-effects models (LME) with a random intercept for melanoma cell line, fitted by restricted maximum likelihood (REML). Adhesion state (adherent vs. non-adherent) and melanoma origin (metastatic vs. primary) were modelled as categorical fixed effects. Endothelial origin was encoded as a set of indicator variables. Two complementary modelling strategies were applied.

S1.2.1 Context-specific models

Separate models were fit within strata defined by a single experimental factor (endothelial origin, melanoma origin, or adhesion state). For adhesion-effect estimation within endothelial stratum e, the model for gene g and observation i took the form:

$$y_{g,e,i} = \beta_{0g}^{e} + \beta_{1g}^{e} A_i + \beta_{2g}^{e} S_i + b_{c(i)} + \varepsilon_{g,e,i} \qquad (2)$$

where $y_{g,e,i}$ denotes $\log_2$-transformed expression of gene g in endothelial context e; A_i encodes adhesion state (0 = adherent, 1 = non-adherent); S_i encodes melanoma origin (0 = primary, 1 = metastatic); and $b_{c(i)}$ denotes a random intercept for the melanoma cell line c from which observation i derives, with $b_c \sim N(0, \sigma_b^2)$ and $\varepsilon_{g,e,i} \sim N(0, \sigma^2)$.

Analogous context-specific models were fit within strata of melanoma origin (replacing S_i with endothelial-origin indicators O_i) and within strata of adhesion state (replacing A_i with O_i). The adhesion coefficient was sign-flipped to report effects in the direction adherent minus non-adherent. Higher-order interaction terms and random slopes were omitted to

https://doi.org/10.3390/biomedicines14071409

avoid unstable variance-component estimates given the limited number of melanoma cell lines per condition.

S1.2.2 Marginal models

Marginal models estimated average effects by pooling observations over all endothelial origins:

$$y_{g,i} = \beta_{0g} + \beta_{1g}A_i + \beta_{2g}S_i + \beta_{3g}^{T}O_i + b_{c(i)} + \varepsilon_{g,i} \quad (3)$$

where O_i denotes a vector of indicator variables encoding endothelial origin (pulmonary, brain, hepatic), and all other terms are defined as in Eq. (2). The random structure remained a single random intercept for cell line.

S1.2.3 Estimation and convergence

All LME models were fitted using the `statsmodels` implementation of the profile-likelihood approach. REML was used for all final inference to obtain less biased variance-component estimates. A sequential optimizer protocol was applied: L-BFGS was attempted first; if convergence failed, conjugate gradient and Powell's method were applied in succession. Models for which all three optimizers failed were flagged and excluded from downstream inference.

S1.3 Multiple comparison correction

FDR was controlled by the two-stage adaptive Benjamini–Hochberg procedure. For context-specific models, q-values were computed independently within each stratum (i.e., within each endothelial origin, melanoma origin, or adhesion-state subset). For marginal models, q-values were computed over the full set of tested genes. A global pooled FDR adjustment was additionally performed over all context-specific tests within each effect family as a supplementary robustness check. Genes with $q < 0.10$ were considered statistically significant.

S1.4 Effect size measures

S1.4.1 Standardized regression coefficients

Two complementary effect-size standardizations were computed for each LME contrast. The first divides the fixed-effect estimate by the unconditional (total) standard deviation of the gene's $\log_2$-expression values:

$$d_g^{\text{total}} = \hat{\beta}_g / \text{SD}_g \quad (4)$$

providing a unit-free metric comparable in scale to a correlation coefficient. The second divides by the model residual standard deviation (square root of the estimated residual variance from the LME), yielding a within-group Cohen's d analogue:

$$d_g^{\text{resid}} = \hat{\beta}_g / \hat{\sigma}_\varepsilon \quad (5)$$

S1.4.2 Hedges' g

https://doi.org/10.3390/biomedicines14071409

Unstratified group differences between primary and metastatic melanoma cell lines were quantified by Hedges' g, the bias-corrected standardized mean difference:

$$g = J \cdot (\bar{x}_P - \bar{x}_M) / s_p \quad (6)$$

where s_p denotes the pooled standard deviation, $s_p = \sqrt{[((n_P - 1)s_P^2 + (n_M - 1)s_M^2) / (n_P + n_M - 2)]}$, and $J = 1 - 3 / (4(n_P + n_M) - 9)$ is the small-sample bias correction factor. Positive values of g denote higher expression in primary cell lines.

S1.5 Adhesion-score analysis

Differential organotropic adhesion was quantified as pairwise differences in $\log_2$-transformed adhesion scores relative to the hepatic endothelium (brain minus hepatic; pulmonary minus hepatic). For each melanoma-origin group (primary, metastatic), the group mean and its 95% confidence interval were computed using the Student t-distribution:

$$\bar{x} \pm t_{n-1, 0.975} \cdot s / \sqrt{n} \quad (7)$$

where n is the number of cell lines per group and s is the sample standard deviation of the delta values.

S1.6 Gene–adhesion Spearman correlations

For each endothelial context $e \in$ {hepatic, brain, pulmonary}, the monotonic association between individual gene expression and endothelial-specific adhesion scores was assessed by Spearman's rank correlation coefficient:

$$\rho_g^e = \mathrm{corr}(\mathrm{R}(x_g^e), \mathrm{R}(a^e)) \quad (8)$$

where x_g^e is the vector of $\log_2$-expression values for gene g and a^e is the vector of adhesion scores, both within endothelial context e, and $\mathrm{R}(\cdot)$ denotes ranks. Correlations were computed only for gene–context pairs with $n \geq 4$ complete observations. The top 25 genes by maximum absolute $|\rho|$ were retained for visualization.

S1.7 Gene–gene coordination analysis

Gene–gene coordination was quantified within each endothelial context by pairwise Spearman rank correlation computed on condition-level expression profiles (cell line × adhesion state × melanoma origin). For each endothelial context e, expression data were pivoted to a wide matrix with rows indexing condition-level samples and columns indexing genes. The Spearman correlation matrix was computed as:

$$\rho_{gi,gj}^e = \mathrm{corr}(\mathrm{R}(x_{gi}^e), \mathrm{R}(x_{gj}^e)) \quad (9)$$

where x_g^e is the vector of $\log_2$-expression values for gene g over all condition-level samples within endothelial context e. Coordination strength was summarized as the mean absolute upper-triangular correlation, $\langle|\rho|\rangle$.

https://doi.org/10.3390/biomedicines14071409

To assess whether observed coordination exceeded chance expectation, permutation testing was conducted independently within each endothelial context. In each permutation (B = 200), gene expression values were shuffled independently within each sample (row), thereby destroying gene–gene dependencies while preserving the sample-level marginal distributions. The full Spearman correlation matrix was recomputed on each permuted dataset, and the mean absolute upper-triangular correlation was recorded. The resulting null distribution of $\langle|\varrho|\rangle$ was summarized by its median and 95% interval (2.5th and 97.5th percentiles). The observed $\langle|\varrho|\rangle$ was compared visually and numerically against this null interval.

Stratified heatmaps were additionally constructed by computing Spearman correlation matrices within each organ-by-stratum subset (adhesion state or melanoma origin) and averaging the resulting matrices over organs.

S1.8 qRT-PCR dataset for melanoma tissue samples

Differential expression between primary and metastatic melanoma tissue samples in the independent qRT-PCR validation dataset was estimated using gene-wise linear mixed-effects models with melanoma origin as the sole fixed effect and a random intercept for sample identity to account for replicate measurements:

$$y_{g,i} = \beta_{0g} + \beta_{1g}G_i + b_{s(i)} + \varepsilon_{g,i} \tag{10}$$

where $y_{g,i}$ is the $\log_2$-transformed expression of gene g in replicate i; G_i is a binary indicator for metastatic origin (0 = primary, 1 = metastatic); and $b_{s(i)}$ is a random intercept for the sample (cell line) from which replicate i derives. Models were fitted by REML. The coefficient $\hat{\beta}_{1g}$ estimates the $\log_2$ fold change (metastatic relative to primary); fold change on the natural scale was recovered as $2^{\hat{\beta}}$. Replicate-level observations (R1, R2) were individually $\log_2$-transformed prior to model fitting. Multiple testing correction was performed by two-stage Benjamini–Hochberg ($q < 0.10$).

S1.9 Software and visualization

All analyses were implemented in Python 3.11. Data manipulation: pandas (v2.x), NumPy. Statistical modelling: statsmodels (mixed-effects models, Benjamini–Hochberg and two-stage BH corrections), SciPy (Spearman correlations, hierarchical clustering, spline fitting for Storey π_0 estimation). Imputation: scikit-learn (KNNImputer). Visualization: matplotlib, seaborn. Final figure assembly and annotation were performed in GraphPad Prism (v10.6).

https://doi.org/10.3390/biomedicines14071409

2. Supplementary Figure S1: Gene expression differences between metastatic and primary melanoma cell lines

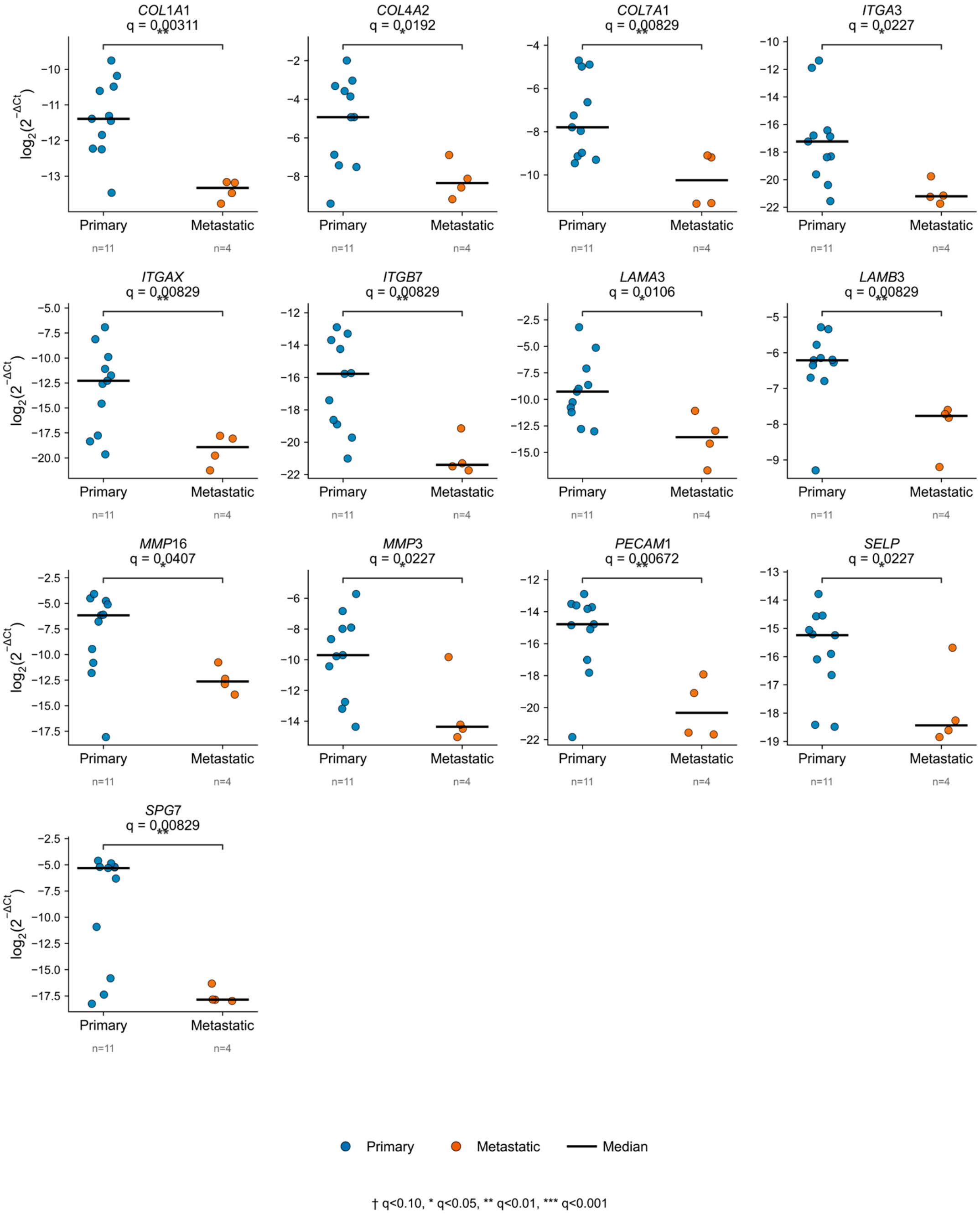

Supplementary Figure S1. Strip plots of individual melanoma cell lines expression values for significant genes, 13 genes meet the FDR threshold of q ≤ 0.10. Horizontal black lines denote group medians. Blue: primary; red: metastatic. Per-group sample sizes are annotated below each strip. * *q* < 0.05, ** *q* < 0.01.

3. Supplementary Figure S2: Gene expression differences between metastatic and primary melanoma tissue samples

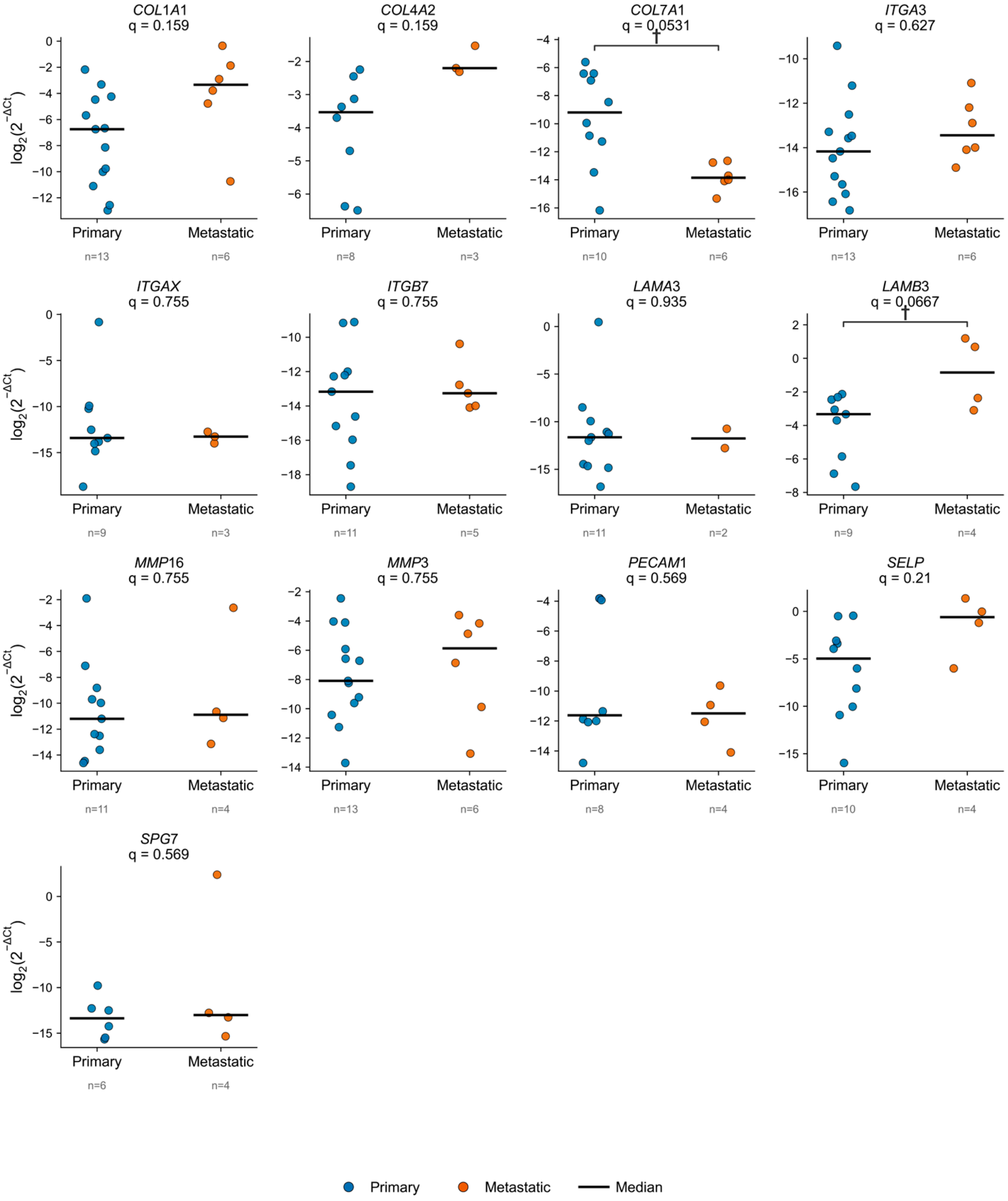

Supplementary Figure S2. Strip plots of individual melanoma tissue samples expression values for 13 tested genes. *COL7A1* and *LAMB3* meet the FDR threshold of $q \leq 0.10$. Horizontal black lines denote group medians. Blue: primary; red: metastatic. Per-group sample sizes are annotated below each strip. † $q < 0.1$.

https://doi.org/10.3390/biomedicines14071409

4. Supplementary Figure S3: Representative fluorescence microscopic images of adherent melanoma cells

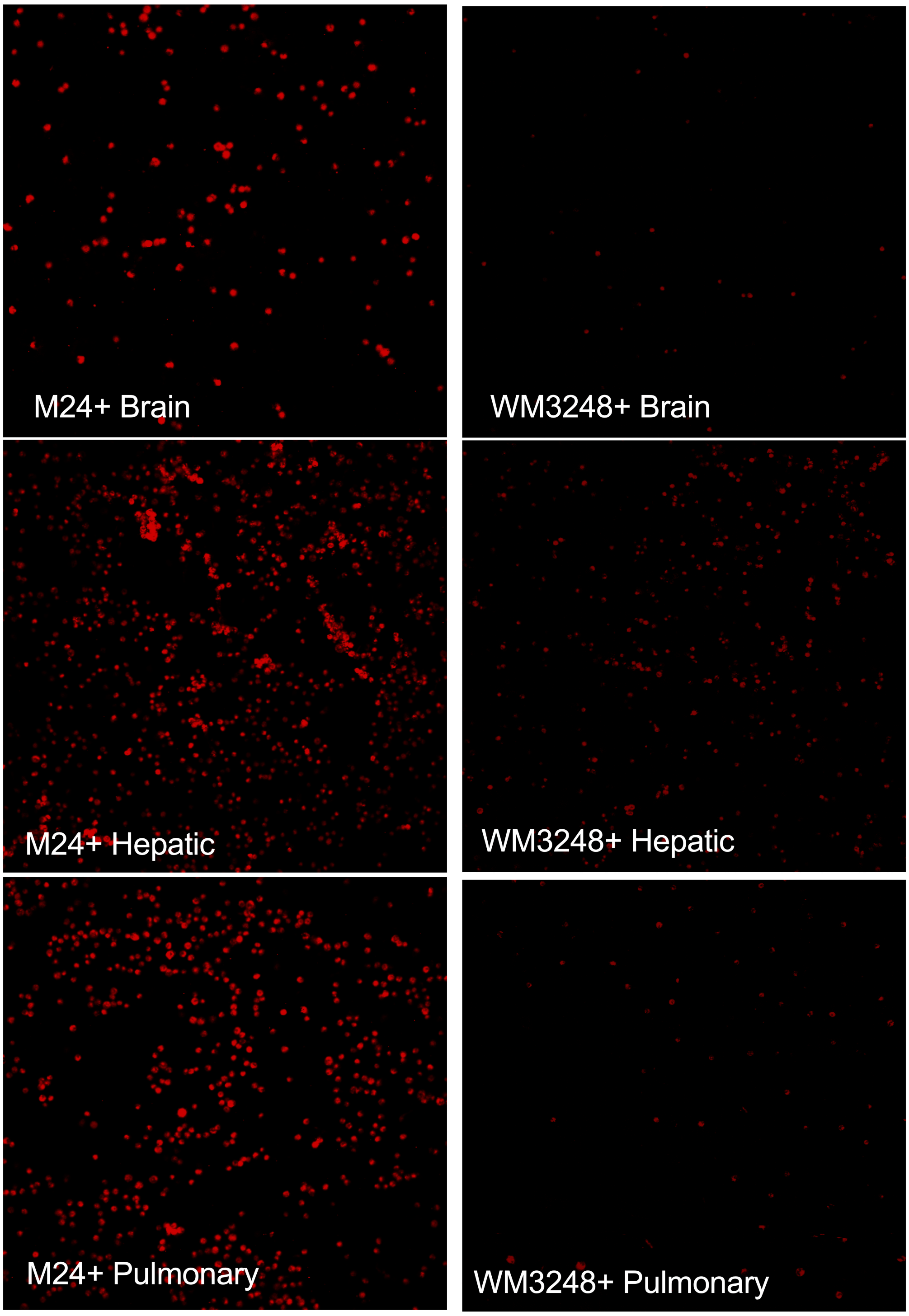

Supplementary Figure S3. Representative images of primary (WM3248) and metastatic (M24) cell lines showing red PKH26 labelled adherent melanoma cells on brain, hepatic, and pulmonary endothelial monolayers.

5. Supplementary Table S1: Clinicopathological characteristics of human melanoma cell lines

Cell line	Growth Phase[1]	Histological Subtype[2]	BRAF Mutation Status[3]	NRAS Mutation Status[4]
Primary tumor derived cell lines				
WM35	RGP	SSM	V600E	WT
WM3211	RGP/VGP	SSM	WT	WT
WM1361	VGP	SSM	WT	Q61L
WM1366	VGP	SSM	WT	Q61L
WM278	VGP	NM	V600E	WT
WM793B	RGP/VGP	SSM	V600E	WT
WM983A	VGP	NM	V600E	WT
WM115	RGP/VGP	SSM	V600D	WT
A375	n.d.	n.d.	V600E	WT
WM3248	VGP	n.d.	V600E	WT
Mel1617	n.d.	n.d.	V600E	WT
Melanoma metastasis originated cell lines				
WM983B	-	-	V600E	WT
A2058	-	-	V600E	WT
M24	-	-	WT	Q61R
WM266-4			V600D	WT

[1] RGP: radial growth phase; VGP: vertical growth phase; [2] SSM: superficial spreading melanoma; NM: nodular melanoma; n.d.: data not available; [3] V: valine; E: glutamic acid; WT: wild type; [4] Q: glutamine; and L: leucine.

6. Supplementary Table S2: Clinical–pathological parameters of the melanoma tumor samples

Variable	No. of tumours analysed	
All patients	20	Primary(n=14), Metastatic (n=6)
Clinical–pathological parameters of primary tumours		
Histological subtype		
SSM[1]	10	
NM[2]	4	
sex		
Female	9	
Male	5	
Age (years)		
20−50	4	
≥50	10	
Berslow thickness (mm)[3]		
≤2.00	7	
2.01−4.00	3	
>4.01	4	
Clark level		
I–III (early)	6	
IV−V (late)	8	
Ulceration		
Absent	6	
Present	8	
Localization		
Extremity	10	
Trunk	4	
Head	0	
Metastasis[4]		
Absent	5	
Present	9	

[1] SSM: superficial spreading melanoma; [2] NM: nodular melanoma; [3] Thickness categories are based on the current staging system; [4] Metastasis of the examined primary tumour.

7. Supplementary Table S3: Marginal model results of gene-level expression effects for adhesion-state adjusted for melanoma-origin and endothelial type

Table lists the standardized effect magnitudes, standard errors, and significance levels (p and q values) for all 86 genes.

Gene	estimate	se	p value	q value
ADAMTS1	1.08	0.41	0.01	0.13
ADAMTS13	0.98	0.50	0.05	0.27
ADAMTS8	0.39	0.45	0.39	0.67
CD44	-0.33	0.37	0.37	0.64
CDH1	0.38	0.36	0.30	0.58
CNTN1	-0.55	0.53	0.30	0.58
COL11A1	-0.54	0.37	0.15	0.42
COL12A1	-0.75	0.25	0.00	0.05
COL14A1	-0.24	0.30	0.42	0.69
COL15A1	-1.97	1.12	0.08	0.32
COL16A1	0.09	0.26	0.72	0.87
COL1A1	0.35	1.62	0.83	0.94
COL4A2	-0.87	0.73	0.23	0.55
COL5A1	1.03	0.95	0.28	0.58
COL6A1	-0.01	0.43	0.98	0.98
COL7A1	-2.52	1.44	0.08	0.32
COL8A1	-1.74	0.92	0.06	0.27
CTGF	0.06	0.42	0.89	0.95
CTNNA1	-0.24	0.25	0.34	0.61
CTNNB1	-0.36	0.22	0.10	0.33
CTNND1	0.01	0.20	0.94	0.97
CTNND2	-0.79	0.68	0.24	0.56
ECM1	0.65	1.66	0.70	0.87
FN1	1.39	0.72	0.05	0.27
HAS1	-1.95	0.54	0.00	0.02
ICAM1	-0.71	0.56	0.20	0.49
ITGA1	0.32	0.81	0.69	0.87
ITGA10	0.99	1.68	0.56	0.81
ITGA11	0.50	0.54	0.36	0.64
ITGA2	-0.14	0.29	0.64	0.87
ITGA3	-0.38	1.07	0.72	0.87
ITGA4	-2.49	1.50	0.10	0.33
ITGA5	0.03	0.09	0.73	0.87
ITGA6	-0.45	0.78	0.56	0.81
ITGA7	-0.42	0.37	0.27	0.58
ITGA8	-1.57	0.93	0.09	0.33

https://doi.org/10.3390/biomedicines14071409

ITGA9	-1.16	0.76	0.13	0.40
ITGAD	0.19	0.36	0.60	0.85
ITGAL	-0.34	0.47	0.46	0.75
ITGAM	0.04	0.50	0.94	0.97
ITGAV	-0.43	0.65	0.50	0.79
ITGAX	-0.63	1.54	0.68	0.87
ITGB1	0.71	0.27	0.01	0.13
ITGB2	0.50	0.69	0.47	0.75
ITGB3	-1.88	1.26	0.14	0.40
ITGB4	-1.40	1.24	0.26	0.57
ITGB5	0.05	0.42	0.90	0.95
ITGB6	-0.57	0.43	0.18	0.47
ITGB7	-0.26	1.17	0.82	0.94
ITGB8	-2.09	0.83	0.01	0.13
LAMA1	-1.52	0.80	0.06	0.27
LAMA2	1.72	1.78	0.33	0.61
LAMA3	0.80	1.74	0.65	0.87
LAMB1	-0.02	0.47	0.96	0.98
LAMB3	-0.91	0.51	0.07	0.32
LAMC1	-0.21	0.97	0.83	0.94
MMP1	0.48	1.22	0.70	0.87
MMP10	-1.41	0.55	0.01	0.13
MMP11	1.05	1.09	0.34	0.61
MMP12	0.08	0.45	0.85	0.95
MMP13	2.34	1.23	0.06	0.27
MMP14	-0.20	0.33	0.54	0.81
MMP15	-1.59	0.82	0.05	0.27
MMP16	-1.29	0.93	0.16	0.45
MMP2	0.01	0.39	0.99	0.98
MMP3	-0.99	0.95	0.30	0.58
MMP7	-1.11	0.47	0.02	0.17
MMP8	-0.32	1.30	0.81	0.94
MMP9	1.44	0.44	0.00	0.05
NCAM1	1.32	2.79	0.64	0.87
PECAM1	1.74	1.32	0.19	0.48
SELE	-0.62	1.68	0.71	0.87
SELL	0.06	0.71	0.93	0.97
SELP	0.43	0.40	0.28	0.58
SPARC	0.05	0.38	0.90	0.95
SPG7	2.17	1.28	0.09	0.33
SPP1	-1.75	0.80	0.03	0.25
TGFB1	0.19	0.33	0.55	0.81

THBS1	0.64	0.98	0.52	0.80
THBS2	-2.07	1.23	0.09	0.33
THBS3	0.74	0.24	0.00	0.05
TIMP1	-0.59	0.39	0.13	0.40
TIMP2	0.33	0.16	0.04	0.27
TNC	-1.38	0.65	0.03	0.26
VCAM1	-0.09	0.53	0.87	0.95
VCAN	-0.90	0.69	0.19	0.48

8. Supplementary Table S4: Marginal model results of gene-level expression effects for melanoma-origin adjusted for adhesion-state and endothelial type

Table lists the standardized effect magnitudes, standard errors, and significance levels (p and q values) for all 86 genes.

Gene	estimate	se	p value	q value
ADAMTS1	-1.36	1.64	0.41	0.81
ADAMTS13	-1.31	1.11	0.24	0.75
ADAMTS8	-0.76	0.84	0.37	0.79
CD44	0.05	1.04	0.96	0.91
CDH1	1.18	4.12	0.77	0.90
CNTN1	2.00	3.89	0.61	0.90
COL11A1	0.38	1.07	0.73	0.90
COL12A1	-1.22	0.80	0.13	0.53
COL14A1	1.70	2.10	0.42	0.81
COL15A1	-0.44	4.60	0.92	0.91
COL16A1	-1.50	1.51	0.32	0.76
COL1A1	-2.25	3.35	0.50	0.90
COL4A2	-0.28	3.37	0.93	0.91
COL5A1	-4.80	2.08	0.02	0.14
COL6A1	-0.32	1.70	0.85	0.90
COL7A1	1.84	4.32	0.67	0.90
COL8A1	-3.85	1.47	0.01	0.08
CTGF	-1.52	0.42	0.00	0.00
CTNNA1	0.24	1.25	0.85	0.90
CTNNB1	-0.16	1.04	0.88	0.90
CTNND1	0.25	0.52	0.63	0.90
CTNND2	1.36	2.50	0.59	0.90
ECM1	3.22	3.20	0.31	0.76
FN1	-2.72	2.26	0.23	0.75
HAS1	-0.18	0.65	0.78	0.90

ICAM1	0.00	1.43	1.00	0.92
ITGA1	-2.52	2.36	0.28	0.76
ITGA10	-1.87	1.78	0.29	0.76
ITGA11	-6.54	1.62	0.00	0.00
ITGA2	2.07	0.94	0.03	0.17
ITGA3	1.14	1.40	0.41	0.81
ITGA4	5.60	3.63	0.12	0.53
ITGA5	-0.64	0.09	0.00	0.00
ITGA6	0.21	1.22	0.86	0.90
ITGA7	2.55	1.84	0.17	0.60
ITGA8	2.19	3.68	0.55	0.90
ITGA9	-1.36	2.63	0.61	0.90
ITGAD	-0.98	0.74	0.19	0.64
ITGAL	-0.51	1.14	0.65	0.90
ITGAM	1.54	1.42	0.28	0.76
ITGAV	-1.86	2.28	0.42	0.81
ITGAX	-2.73	1.68	0.11	0.49
ITGB1	-1.32	0.53	0.01	0.11
ITGB2	-0.05	0.73	0.95	0.91
ITGB3	2.08	2.68	0.44	0.82
ITGB4	-4.72	3.36	0.16	0.60
ITGB5	0.38	0.88	0.66	0.90
ITGB6	-0.36	1.57	0.82	0.90
ITGB7	0.55	0.85	0.52	0.90
ITGB8	5.53	1.96	0.00	0.05
LAMA1	6.43	3.69	0.08	0.40
LAMA2	0.31	1.92	0.87	0.90
LAMA3	-0.68	2.86	0.81	0.90
LAMB1	-0.83	1.53	0.59	0.90
LAMB3	0.46	1.96	0.81	0.90
LAMC1	-0.24	1.22	0.84	0.90
MMP1	-4.84	5.36	0.37	0.79
MMP10	-2.31	0.80	0.00	0.04
MMP11	-0.21	1.09	0.85	0.90
MMP12	-2.07	0.89	0.02	0.14
MMP13	1.80	1.22	0.14	0.55
MMP14	0.10	0.39	0.79	0.90
MMP15	-0.06	3.23	0.98	0.91
MMP16	0.65	1.16	0.58	0.90
MMP2	-4.40	0.39	0.00	0.00
MMP3	-4.87	1.25	0.00	0.00
MMP7	3.48	1.72	0.04	0.24

MMP8	-1.68	2.63	0.52	0.90
MMP9	-1.54	1.67	0.36	0.79
NCAM1	0.89	2.97	0.76	0.90
PECAM1	0.30	1.30	0.82	0.90
SELE	1.49	2.48	0.55	0.90
SELL	-1.33	4.38	0.76	0.90
SELP	-1.09	0.94	0.25	0.75
SPARC	-2.10	1.97	0.29	0.76
SPG7	-0.80	1.28	0.53	0.90
SPP1	9.66	1.87	0.00	0.00
TGFB1	-0.16	0.47	0.74	0.90
THBS1	-5.37	2.68	0.05	0.24
THBS2	0.42	4.53	0.93	0.91
THBS3	-0.02	0.74	0.97	0.91
TIMP1	0.03	1.08	0.97	0.91
TIMP2	0.34	0.37	0.36	0.79
TNC	0.61	2.47	0.80	0.90
VCAM1	-1.05	2.68	0.70	0.90
VCAN	1.97	1.95	0.31	0.76

9. Supplementary Table S5: Results for all 86 tested genes of linear mixed-effects models fitted within each endothelial context to estimate adhesion-state effects on expression, adjusting for melanoma-origin

Gene	Stratum-type	stratum	estimate	se	p value	q value
ADAMTS1	organ	brain	1.23	0.62	0.05	0.29
ADAMTS13	organ	brain	1.23	1.52	0.42	0.66
ADAMTS8	organ	brain	0.16	0.80	0.84	0.84
CD44	organ	brain	-1.07	0.71	0.13	0.42
CDH1	organ	brain	-0.67	0.80	0.40	0.66
CNTN1	organ	brain	-1.83	0.99	0.06	0.33
COL11A1	organ	brain	-0.94	0.80	0.24	0.52
COL12A1	organ	brain	-0.49	0.57	0.40	0.66
COL14A1	organ	brain	-0.26	0.72	0.71	0.84
COL15A1	organ	brain	-2.61	2.92	0.37	0.66
COL16A1	organ	brain	0.14	0.08	0.07	0.33
COL1A1	organ	brain	1.47	0.61	0.02	0.11
COL4A2	organ	brain	-0.27	1.90	0.89	0.84
COL5A1	organ	brain	2.58	0.62	0.00	0.00
COL6A1	organ	brain	-0.04	0.87	0.97	0.89
COL7A1	organ	brain	-1.17	2.07	0.57	0.72
COL8A1	organ	brain	-1.35	1.77	0.45	0.69

https://doi.org/10.3390/biomedicines14071409

CTGF	organ	brain	-0.07	1.76	0.97	0.89
CTNNA1	organ	brain	-0.76	0.62	0.22	0.48
CTNNB1	organ	brain	-0.69	0.43	0.11	0.39
CTNND1	organ	brain	-0.27	0.34	0.41	0.66
CTNND2	organ	brain	-3.06	1.08	0.00	0.05
ECM1	organ	brain	2.31	3.54	0.51	0.69
FN1	organ	brain	1.88	0.04	0.00	0.00
HAS1	organ	brain	-0.79	0.58	0.17	0.42
ICAM1	organ	brain	-1.43	1.39	0.30	0.59
ITGA1	organ	brain	-1.19	1.62	0.47	0.69
ITGA10	organ	brain	2.85	4.18	0.50	0.69
ITGA11	organ	brain	1.05	0.25	0.00	0.00
ITGA2	organ	brain	-0.02	0.16	0.88	0.84
ITGA3	organ	brain	-0.33	0.74	0.66	0.81
ITGA4	organ	brain	-2.38	3.65	0.51	0.69
ITGA5	organ	brain	-0.03	0.11	0.76	0.84
ITGA6	organ	brain	-1.16	0.80	0.14	0.42
ITGA7	organ	brain	-1.23	0.51	0.02	0.11
ITGA8	organ	brain	-0.73	0.45	0.11	0.39
ITGA9	organ	brain	-1.76	2.03	0.39	0.66
ITGAD	organ	brain	0.15	0.73	0.84	0.84
ITGAL	organ	brain	-1.24	0.10	0.00	0.00
ITGAM	organ	brain	0.14	0.82	0.86	0.84
ITGAV	organ	brain	-0.62	1.00	0.54	0.69
ITGAX	organ	brain	1.10	1.16	0.34	0.65
ITGB1	organ	brain	0.84	0.72	0.25	0.52
ITGB2	organ	brain	2.51	1.57	0.11	0.39
ITGB3	organ	brain	-3.13	2.92	0.28	0.57
ITGB4	organ	brain	-3.21	2.52	0.20	0.47
ITGB5	organ	brain	-1.05	0.73	0.15	0.42
ITGB6	organ	brain	-0.57	0.44	0.19	0.46
ITGB7	organ	brain	-1.67	1.15	0.15	0.42
ITGB8	organ	brain	-2.65	1.89	0.16	0.42
LAMA1	organ	brain	-2.26	0.73	0.00	0.02
LAMA2	organ	brain	3.80	2.12	0.07	0.33
LAMA3	organ	brain	0.50	3.17	0.88	0.84
LAMB1	organ	brain	-0.72	0.37	0.05	0.30
LAMB3	organ	brain	-0.61	0.98	0.53	0.69
LAMC1	organ	brain	-0.33	1.33	0.80	0.84
MMP1	organ	brain	0.62	0.99	0.53	0.69
MMP10	organ	brain	-1.03	0.06	0.00	0.00
MMP11	organ	brain	0.96	2.26	0.67	0.81

MMP12	organ	brain	0.27	0.82	0.74	0.84
MMP13	organ	brain	-0.27	1.35	0.84	0.84
MMP14	organ	brain	0.15	0.70	0.83	0.84
MMP15	organ	brain	-2.44	1.92	0.20	0.47
MMP16	organ	brain	-2.69	1.85	0.15	0.42
MMP2	organ	brain	-0.01	0.42	0.97	0.89
MMP3	organ	brain	-1.44	0.84	0.09	0.35
MMP7	organ	brain	-0.21	0.74	0.78	0.84
MMP8	organ	brain	-0.90	2.08	0.66	0.81
MMP9	organ	brain	2.37	0.86	0.01	0.05
NCAM1	organ	brain	0.30	1.01	0.76	0.84
PECAM1	organ	brain	0.25	0.42	0.54	0.69
SELE	organ	brain	1.19	0.67	0.08	0.34
SELL	organ	brain	0.34	1.08	0.75	0.84
SELP	organ	brain	0.63	0.87	0.47	0.69
SPARC	organ	brain	-0.14	0.44	0.75	0.84
SPG7	organ	brain	1.95	0.74	0.01	0.06
SPP1	organ	brain	-1.96	1.42	0.17	0.42
TGFB1	organ	brain	0.83	0.14	0.00	0.00
THBS1	organ	brain	1.58	1.89	0.40	0.66
THBS2	organ	brain	-2.29	3.22	0.48	0.69
THBS3	organ	brain	0.70	0.61	0.25	0.52
TIMP1	organ	brain	-0.90	1.01	0.37	0.66
TIMP2	organ	brain	0.14	NA	NA	NA
TNC	organ	brain	-1.32	1.83	0.47	0.69
VCAM1	organ	brain	0.63	0.44	0.15	0.42
VCAN	organ	brain	-0.48	1.65	0.77	0.84
ADAMTS1	organ	hepatic	1.59	1.08	0.14	0.44
ADAMTS13	organ	hepatic	1.11	0.31	0.00	0.01
ADAMTS8	organ	hepatic	0.10	0.45	0.83	0.88
CD44	organ	hepatic	-0.32	0.76	0.67	0.82
CDH1	organ	hepatic	0.36	0.08	0.00	0.00
CNTN1	organ	hepatic	-0.59	1.16	0.61	0.78
COL11A1	organ	hepatic	-0.58	0.77	0.45	0.77
COL12A1	organ	hepatic	-0.30	0.21	0.15	0.44
COL14A1	organ	hepatic	-0.29	0.51	0.57	0.78
COL15A1	organ	hepatic	-2.07	3.32	0.53	0.78
COL16A1	organ	hepatic	0.55	0.45	0.22	0.53
COL1A1	organ	hepatic	-0.56	0.76	0.46	0.77
COL4A2	organ	hepatic	-0.92	1.61	0.57	0.78
COL5A1	organ	hepatic	1.50	0.89	0.09	0.39
COL6A1	organ	hepatic	1.14	0.67	0.09	0.39

COL7A1	organ	hepatic	-5.24	2.96	0.08	0.39
COL8A1	organ	hepatic	-2.32	1.58	0.14	0.44
CTGF	organ	hepatic	0.10	0.95	0.91	0.89
CTNNA1	organ	hepatic	-0.03	0.66	0.97	0.91
CTNNB1	organ	hepatic	-0.37	0.60	0.54	0.78
CTNND1	organ	hepatic	0.06	0.57	0.92	0.89
CTNND2	organ	hepatic	-0.22	0.58	0.71	0.84
ECM1	organ	hepatic	-1.94	2.82	0.49	0.77
FN1	organ	hepatic	0.74	1.06	0.49	0.77
HAS1	organ	hepatic	-0.90	0.57	0.11	0.40
ICAM1	organ	hepatic	-0.56	1.42	0.69	0.84
ITGA1	organ	hepatic	2.48	0.97	0.01	0.12
ITGA10	organ	hepatic	-0.75	2.35	0.75	0.84
ITGA11	organ	hepatic	-0.38	1.10	0.73	0.84
ITGA2	organ	hepatic	0.26	0.47	0.58	0.78
ITGA3	organ	hepatic	-2.93	2.52	0.25	0.54
ITGA4	organ	hepatic	-4.27	3.34	0.20	0.51
ITGA5	organ	hepatic	0.11	0.16	0.49	0.77
ITGA6	organ	hepatic	-0.87	0.72	0.23	0.53
ITGA7	organ	hepatic	-0.84	0.52	0.11	0.40
ITGA8	organ	hepatic	-5.00	1.49	0.00	0.01
ITGA9	organ	hepatic	-1.42	2.01	0.48	0.77
ITGAD	organ	hepatic	-0.18	0.57	0.75	0.84
ITGAL	organ	hepatic	-0.27	1.19	0.82	0.88
ITGAM	organ	hepatic	-0.24	0.48	0.61	0.78
ITGAV	organ	hepatic	-0.62	0.53	0.24	0.54
ITGAX	organ	hepatic	-2.78	2.15	0.20	0.51
ITGB1	organ	hepatic	1.08	0.46	0.02	0.14
ITGB2	organ	hepatic	-0.18	0.55	0.74	0.84
ITGB3	organ	hepatic	-1.54	2.86	0.59	0.78
ITGB4	organ	hepatic	-1.92	1.11	0.08	0.39
ITGB5	organ	hepatic	0.26	0.58	0.65	0.81
ITGB6	organ	hepatic	-0.73	0.79	0.35	0.73
ITGB7	organ	hepatic	-0.50	0.31	0.10	0.40
ITGB8	organ	hepatic	-2.25	1.79	0.21	0.51
LAMA1	organ	hepatic	-2.36	1.27	0.06	0.39
LAMA2	organ	hepatic	2.28	1.52	0.13	0.44
LAMA3	organ	hepatic	-0.32	2.76	0.91	0.89
LAMB1	organ	hepatic	-0.14	0.80	0.86	0.89
LAMB3	organ	hepatic	-1.88	1.08	0.08	0.39
LAMC1	organ	hepatic	-0.67	0.93	0.47	0.77
MMP1	organ	hepatic	2.05	1.59	0.20	0.51

MMP10	organ	hepatic	-0.94	0.59	0.11	0.40
MMP11	organ	hepatic	1.46	1.68	0.39	0.73
MMP12	organ	hepatic	0.23	1.16	0.84	0.88
MMP13	organ	hepatic	0.44	0.52	0.39	0.73
MMP14	organ	hepatic	-0.79	0.31	0.01	0.12
MMP15	organ	hepatic	-1.86	2.15	0.39	0.73
MMP16	organ	hepatic	1.77	1.39	0.20	0.51
MMP2	organ	hepatic	0.65	0.23	0.00	0.07
MMP3	organ	hepatic	-0.52	1.92	0.79	0.86
MMP7	organ	hepatic	-2.24	0.67	0.00	0.01
MMP8	organ	hepatic	-2.34	0.94	0.01	0.12
MMP9	organ	hepatic	0.98	1.08	0.37	0.73
NCAM1	organ	hepatic	2.41	4.68	0.61	0.78
PECAM1	organ	hepatic	4.52	0.34	0.00	0.00
SELE	organ	hepatic	0.29	2.60	0.91	0.89
SELL	organ	hepatic	-1.77	1.35	0.19	0.51
SELP	organ	hepatic	0.07	0.95	0.94	0.89
SPARC	organ	hepatic	-0.22	1.07	0.84	0.88
SPG7	organ	hepatic	-0.14	0.48	0.77	0.86
SPP1	organ	hepatic	-3.01	1.53	0.05	0.36
TGFB1	organ	hepatic	0.75	0.43	0.08	0.39
THBS1	organ	hepatic	1.24	1.96	0.53	0.78
THBS2	organ	hepatic	-2.81	3.32	0.40	0.73
THBS3	organ	hepatic	1.02	0.53	0.06	0.38
TIMP1	organ	hepatic	-0.61	0.98	0.53	0.78
TIMP2	organ	hepatic	0.26	0.30	0.38	0.73
TNC	organ	hepatic	-1.59	1.91	0.40	0.73
VCAM1	organ	hepatic	-0.45	0.85	0.60	0.78
VCAN	organ	hepatic	-0.21	1.64	0.90	0.89
ADAMTS1	organ	pulmonary	0.33	0.91	0.71	0.72
ADAMTS13	organ	pulmonary	0.28	0.64	0.66	0.70
ADAMTS8	organ	pulmonary	0.82	0.12	0.00	0.00
CD44	organ	pulmonary	0.48	0.64	0.45	0.60
CDH1	organ	pulmonary	1.45	0.32	0.00	0.00
CNTN1	organ	pulmonary	0.78	0.69	0.26	0.57
COL11A1	organ	pulmonary	-0.02	0.36	0.96	0.85
COL12A1	organ	pulmonary	-1.22	0.37	0.00	0.01
COL14A1	organ	pulmonary	-0.17	0.44	0.70	0.72
COL15A1	organ	pulmonary	-0.69	0.67	0.31	0.60
COL16A1	organ	pulmonary	-0.42	0.32	0.19	0.52
COL1A1	organ	pulmonary	-0.86	1.28	0.50	0.63
COL4A2	organ	pulmonary	-1.17	0.55	0.03	0.20

COL5A1	organ	pulmonary	-0.66	0.75	0.38	0.60
COL6A1	organ	pulmonary	-1.16	0.30	0.00	0.00
COL7A1	organ	pulmonary	-0.43	0.80	0.59	0.66
COL8A1	organ	pulmonary	-1.09	0.88	0.22	0.52
CTGF	organ	pulmonary	0.26	0.34	0.44	0.60
CTNNA1	organ	pulmonary	0.09	0.30	0.77	0.76
CTNNB1	organ	pulmonary	0.04	0.38	0.91	0.83
CTNND1	organ	pulmonary	0.17	0.32	0.58	0.66
CTNND2	organ	pulmonary	1.03	0.38	0.01	0.05
ECM1	organ	pulmonary	2.03	1.28	0.11	0.43
FN1	organ	pulmonary	1.57	1.88	0.40	0.60
HAS1	organ	pulmonary	-4.18	0.82	0.00	0.00
ICAM1	organ	pulmonary	0.17	0.19	0.36	0.60
ITGA1	organ	pulmonary	-0.26	0.49	0.60	0.67
ITGA10	organ	pulmonary	0.29	0.23	0.22	0.52
ITGA11	organ	pulmonary	0.83	1.06	0.44	0.60
ITGA2	organ	pulmonary	-0.56	0.36	0.11	0.43
ITGA3	organ	pulmonary	2.08	1.45	0.15	0.49
ITGA4	organ	pulmonary	0.83	1.06	0.44	0.60
ITGA5	organ	pulmonary	0.01	0.03	0.75	0.75
ITGA6	organ	pulmonary	0.23	0.29	0.44	0.60
ITGA7	organ	pulmonary	0.79	0.56	0.16	0.49
ITGA8	organ	pulmonary	1.13	0.81	0.16	0.49
ITGA9	organ	pulmonary	0.22	0.18	0.23	0.52
ITGAD	organ	pulmonary	0.38	0.80	0.64	0.69
ITGAL	organ	pulmonary	0.30	0.25	0.23	0.52
ITGAM	organ	pulmonary	0.14	0.70	0.84	0.78
ITGAV	organ	pulmonary	-0.04	0.69	0.95	0.85
ITGAX	organ	pulmonary	-0.39	1.96	0.84	0.78
ITGB1	organ	pulmonary	0.09	0.15	0.54	0.65
ITGB2	organ	pulmonary	-0.87	0.46	0.06	0.29
ITGB3	organ	pulmonary	-0.29	0.44	0.51	0.63
ITGB4	organ	pulmonary	1.05	0.68	0.12	0.43
ITGB5	organ	pulmonary	0.95	0.13	0.00	0.00
ITGB6	organ	pulmonary	-0.17	1.09	0.88	0.81
ITGB7	organ	pulmonary	-0.98	1.02	0.34	0.60
ITGB8	organ	pulmonary	-0.39	0.44	0.37	0.60
LAMA1	organ	pulmonary	0.12	0.10	0.23	0.52
LAMA2	organ	pulmonary	-1.22	0.74	0.10	0.43
LAMA3	organ	pulmonary	2.89	2.17	0.18	0.52
LAMB1	organ	pulmonary	0.76	0.75	0.31	0.60
LAMB3	organ	pulmonary	-0.16	0.76	0.83	0.78

LAMC1	organ	pulmonary	0.55	0.97	0.57	0.66
MMP1	organ	pulmonary	-1.21	1.39	0.38	0.60
MMP10	organ	pulmonary	-2.14	1.09	0.05	0.29
MMP11	organ	pulmonary	0.91	0.59	0.12	0.43
MMP12	organ	pulmonary	0.44	0.79	0.57	0.66
MMP13	organ	pulmonary	6.78	0.58	0.00	0.00
MMP14	organ	pulmonary	-0.07	0.34	0.84	0.78
MMP15	organ	pulmonary	0.20	0.54	0.71	0.72
MMP16	organ	pulmonary	-2.63	0.92	0.00	0.03
MMP2	organ	pulmonary	-0.65	0.59	0.27	0.58
MMP3	organ	pulmonary	-1.15	1.37	0.40	0.60
MMP7	organ	pulmonary	-0.77	0.45	0.09	0.41
MMP8	organ	pulmonary	2.20	3.36	0.51	0.63
MMP9	organ	pulmonary	0.50	0.76	0.51	0.63
NCAM1	organ	pulmonary	1.39	1.68	0.41	0.60
PECAM1	organ	pulmonary	2.39	2.46	0.33	0.60
SELE	organ	pulmonary	-0.94	2.00	0.64	0.69
SELL	organ	pulmonary	1.60	1.51	0.29	0.59
SELP	organ	pulmonary	0.54	0.57	0.35	0.60
SPARC	organ	pulmonary	0.56	0.03	0.00	0.00
SPG7	organ	pulmonary	3.34	3.11	0.28	0.59
SPP1	organ	pulmonary	0.65	0.78	0.40	0.60
TGFB1	organ	pulmonary	-0.64	0.64	0.32	0.60
THBS1	organ	pulmonary	-0.98	0.52	0.06	0.29
THBS2	organ	pulmonary	-0.59	0.61	0.33	0.60
THBS3	organ	pulmonary	0.16	0.29	0.57	0.66
TIMP1	organ	pulmonary	0.04	0.15	0.78	0.76
TIMP2	organ	pulmonary	0.37	0.29	0.20	0.52
TNC	organ	pulmonary	-0.54	0.35	0.12	0.43
VCAM1	organ	pulmonary	-0.47	0.65	0.46	0.61
VCAN	organ	pulmonary	-1.68	0.30	0.00	0.00
ADAMTS1	melanoma_origin	metastatic	0.67	0.48	0.16	0.60
ADAMTS13	melanoma_origin	metastatic	0.84	0.60	0.16	0.60
ADAMTS8	melanoma_origin	metastatic	0.14	0.95	0.88	0.96
CD44	melanoma_origin	metastatic	0.17	0.75	0.82	0.95
CDH1	melanoma_origin	metastatic	-0.21	0.62	0.74	0.91
CNTN1	melanoma_origin	metastatic	1.07	0.55	0.05	0.35
COL11A1	melanoma_origin	metastatic	0.32	0.50	0.52	0.84
COL12A1	melanoma_origin	metastatic	-1.34	0.49	0.01	0.16
COL14A1	melanoma_origin	metastatic	0.12	0.46	0.80	0.95
COL15A1	melanoma_origin	metastatic	0.64	0.73	0.38	0.81
COL16A1	melanoma_origin	metastatic	0.53	0.55	0.34	0.79

COL1A1	melanoma_origin	metastatic	0.24	2.68	0.93	0.98
COL4A2	melanoma_origin	metastatic	0.26	1.20	0.83	0.95
COL5A1	melanoma_origin	metastatic	0.25	1.68	0.88	0.96
COL6A1	melanoma_origin	metastatic	0.00	0.70	1.00	0.98
COL7A1	melanoma_origin	metastatic	-2.51	2.29	0.27	0.76
COL8A1	melanoma_origin	metastatic	-0.02	1.44	0.99	0.98
CTGF	melanoma_origin	metastatic	0.42	0.53	0.43	0.81
CTNNA1	melanoma_origin	metastatic	0.55	0.36	0.13	0.59
CTNNB1	melanoma_origin	metastatic	0.45	0.32	0.16	0.60
CTNND1	melanoma_origin	metastatic	0.61	0.27	0.03	0.28
CTNND2	melanoma_origin	metastatic	-1.19	1.16	0.31	0.76
ECM1	melanoma_origin	metastatic	1.55	2.39	0.52	0.84
FN1	melanoma_origin	metastatic	1.55	1.01	0.12	0.59
HAS1	melanoma_origin	metastatic	-1.84	0.71	0.01	0.16
ICAM1	melanoma_origin	metastatic	0.95	0.38	0.01	0.16
ITGA1	melanoma_origin	metastatic	0.95	0.94	0.31	0.76
ITGA10	melanoma_origin	metastatic	-0.77	2.21	0.73	0.91
ITGA11	melanoma_origin	metastatic	1.82	0.69	0.01	0.16
ITGA2	melanoma_origin	metastatic	0.39	0.53	0.45	0.81
ITGA3	melanoma_origin	metastatic	0.91	1.73	0.60	0.87
ITGA4	melanoma_origin	metastatic	0.79	1.49	0.60	0.87
ITGA5	melanoma_origin	metastatic	0.11	0.11	0.31	0.76
ITGA6	melanoma_origin	metastatic	1.08	1.35	0.42	0.81
ITGA7	melanoma_origin	metastatic	-0.33	0.55	0.55	0.85
ITGA8	melanoma_origin	metastatic	-1.45	1.48	0.33	0.79
ITGA9	melanoma_origin	metastatic	-0.10	0.28	0.72	0.91
ITGAD	melanoma_origin	metastatic	1.24	0.41	0.00	0.09
ITGAL	melanoma_origin	metastatic	-0.49	0.73	0.51	0.84
ITGAM	melanoma_origin	metastatic	0.87	1.25	0.48	0.83
ITGAV	melanoma_origin	metastatic	0.54	0.68	0.42	0.81
ITGAX	melanoma_origin	metastatic	-1.09	2.28	0.63	0.89
ITGB1	melanoma_origin	metastatic	0.29	0.25	0.24	0.76
ITGB2	melanoma_origin	metastatic	0.67	1.66	0.68	0.91
ITGB3	melanoma_origin	metastatic	0.92	0.84	0.27	0.76
ITGB4	melanoma_origin	metastatic	-1.28	2.04	0.53	0.84
ITGB5	melanoma_origin	metastatic	0.32	0.72	0.66	0.90
ITGB6	melanoma_origin	metastatic	-1.01	0.73	0.17	0.60
ITGB7	melanoma_origin	metastatic	1.22	1.18	0.30	0.76
ITGB8	melanoma_origin	metastatic	-0.60	0.36	0.09	0.55
LAMA1	melanoma_origin	metastatic	-1.12	1.38	0.42	0.81
LAMA2	melanoma_origin	metastatic	1.32	2.55	0.60	0.87
LAMA3	melanoma_origin	metastatic	3.65	2.44	0.13	0.59

LAMB1	melanoma_origin	metastatic	0.65	0.54	0.23	0.75
LAMB3	melanoma_origin	metastatic	0.26	0.66	0.69	0.91
LAMC1	melanoma_origin	metastatic	1.30	0.60	0.03	0.28
MMP1	melanoma_origin	metastatic	-0.05	2.67	0.99	0.98
MMP10	melanoma_origin	metastatic	-1.26	0.94	0.18	0.61
MMP11	melanoma_origin	metastatic	3.83	NA	NA	NA
MMP12	melanoma_origin	metastatic	0.12	0.72	0.87	0.96
MMP13	melanoma_origin	metastatic	2.65	1.95	0.17	0.61
MMP14	melanoma_origin	metastatic	-0.30	0.51	0.56	0.85
MMP15	melanoma_origin	metastatic	0.10	0.69	0.89	0.96
MMP16	melanoma_origin	metastatic	-1.14	1.45	0.43	0.81
MMP2	melanoma_origin	metastatic	-0.42	0.46	0.35	0.79
MMP3	melanoma_origin	metastatic	-0.18	0.62	0.77	0.94
MMP7	melanoma_origin	metastatic	-1.07	0.51	0.04	0.31
MMP8	melanoma_origin	metastatic	3.66	1.99	0.07	0.43
MMP9	melanoma_origin	metastatic	2.30	0.47	0.00	0.00
NCAM1	melanoma_origin	metastatic	-1.25	5.39	0.82	0.95
PECAM1	melanoma_origin	metastatic	0.10	1.76	0.96	0.98
SELE	melanoma_origin	metastatic	-0.13	3.56	0.97	0.98
SELL	melanoma_origin	metastatic	-0.22	0.46	0.63	0.89
SELP	melanoma_origin	metastatic	0.39	0.37	0.30	0.76
SPARC	melanoma_origin	metastatic	0.81	0.40	0.04	0.31
SPG7	melanoma_origin	metastatic	1.58	1.01	0.12	0.59
SPP1	melanoma_origin	metastatic	-0.66	0.72	0.36	0.79
TGFB1	melanoma_origin	metastatic	0.37	0.49	0.45	0.81
THBS1	melanoma_origin	metastatic	0.20	0.50	0.68	0.91
THBS2	melanoma_origin	metastatic	0.54	1.47	0.71	0.91
THBS3	melanoma_origin	metastatic	0.71	0.32	0.03	0.28
TIMP1	melanoma_origin	metastatic	0.28	0.36	0.44	0.81
TIMP2	melanoma_origin	metastatic	0.36	0.23	0.12	0.59
TNC	melanoma_origin	metastatic	0.35	0.50	0.49	0.83
VCAM1	melanoma_origin	metastatic	-0.06	1.18	0.96	0.98
VCAN	melanoma_origin	metastatic	-0.07	0.46	0.87	0.96
ADAMTS1	melanoma_origin	primary	1.29	0.67	0.05	0.17
ADAMTS13	melanoma_origin	primary	1.15	0.79	0.14	0.30
ADAMTS8	melanoma_origin	primary	0.58	0.40	0.15	0.30
CD44	melanoma_origin	primary	-0.62	0.37	0.10	0.23
CDH1	melanoma_origin	primary	0.67	0.36	0.06	0.17
CNTN1	melanoma_origin	primary	-1.36	0.59	0.02	0.16
COL11A1	melanoma_origin	primary	-1.03	0.52	0.05	0.17
COL12A1	melanoma_origin	primary	-0.46	0.21	0.03	0.16
COL14A1	melanoma_origin	primary	-0.43	0.34	0.21	0.36

https://doi.org/10.3390/biomedicines14071409

COL15A1	melanoma_origin	primary	-3.36	1.76	0.06	0.17
COL16A1	melanoma_origin	primary	-0.13	0.21	0.52	0.67
COL1A1	melanoma_origin	primary	0.22	2.29	0.92	0.94
COL4A2	melanoma_origin	primary	-1.47	1.02	0.15	0.30
COL5A1	melanoma_origin	primary	1.52	0.54	0.01	0.11
COL6A1	melanoma_origin	primary	-0.01	0.64	0.99	0.98
COL7A1	melanoma_origin	primary	-2.58	1.33	0.05	0.17
COL8A1	melanoma_origin	primary	-2.99	0.92	0.00	0.05
CTGF	melanoma_origin	primary	-0.32	0.65	0.62	0.73
CTNNA1	melanoma_origin	primary	-0.66	0.30	0.03	0.16
CTNNB1	melanoma_origin	primary	-0.77	0.21	0.00	0.03
CTNND1	melanoma_origin	primary	-0.29	0.25	0.24	0.39
CTNND2	melanoma_origin	primary	-0.59	0.97	0.54	0.68
ECM1	melanoma_origin	primary	0.07	2.30	0.98	0.98
FN1	melanoma_origin	primary	1.33	0.87	0.12	0.27
HAS1	melanoma_origin	primary	-2.17	0.87	0.01	0.15
ICAM1	melanoma_origin	primary	-1.58	0.81	0.05	0.17
ITGA1	melanoma_origin	primary	0.00	1.27	1.00	0.98
ITGA10	melanoma_origin	primary	2.70	2.53	0.28	0.43
ITGA11	melanoma_origin	primary	-0.13	0.62	0.83	0.90
ITGA2	melanoma_origin	primary	-0.42	0.21	0.05	0.17
ITGA3	melanoma_origin	primary	-1.31	1.61	0.41	0.57
ITGA4	melanoma_origin	primary	-4.52	2.20	0.04	0.17
ITGA5	melanoma_origin	primary	-0.04	0.14	0.75	0.85
ITGA6	melanoma_origin	primary	-1.41	0.54	0.01	0.12
ITGA7	melanoma_origin	primary	-0.45	0.51	0.37	0.53
ITGA8	melanoma_origin	primary	-1.65	1.30	0.21	0.36
ITGA9	melanoma_origin	primary	-1.74	1.35	0.20	0.36
ITGAD	melanoma_origin	primary	-0.33	0.42	0.44	0.59
ITGAL	melanoma_origin	primary	-0.25	0.69	0.71	0.82
ITGAM	melanoma_origin	primary	-0.46	0.17	0.01	0.11
ITGAV	melanoma_origin	primary	-0.96	0.67	0.16	0.31
ITGAX	melanoma_origin	primary	-0.28	1.56	0.86	0.91
ITGB1	melanoma_origin	primary	1.00	0.45	0.02	0.16
ITGB2	melanoma_origin	primary	0.29	0.43	0.50	0.64
ITGB3	melanoma_origin	primary	-3.59	1.82	0.05	0.17
ITGB4	melanoma_origin	primary	-1.56	1.46	0.28	0.43
ITGB5	melanoma_origin	primary	-0.08	0.57	0.88	0.92
ITGB6	melanoma_origin	primary	-0.36	0.51	0.49	0.64
ITGB7	melanoma_origin	primary	-1.74	0.76	0.02	0.16
ITGB8	melanoma_origin	primary	-2.90	1.41	0.04	0.17
LAMA1	melanoma_origin	primary	-1.74	0.96	0.07	0.17

LAMA2	melanoma_origin	primary	2.18	1.86	0.24	0.39
LAMA3	melanoma_origin	primary	-1.13	2.14	0.60	0.72
LAMB1	melanoma_origin	primary	-0.35	0.68	0.61	0.72
LAMB3	melanoma_origin	primary	-1.54	0.69	0.03	0.16
LAMC1	melanoma_origin	primary	-1.34	1.23	0.28	0.43
MMP1	melanoma_origin	primary	0.76	0.85	0.37	0.53
MMP10	melanoma_origin	primary	-1.58	0.72	0.03	0.16
MMP11	melanoma_origin	primary	-1.66	1.14	0.14	0.30
MMP12	melanoma_origin	primary	0.06	0.71	0.93	0.94
MMP13	melanoma_origin	primary	2.04	1.49	0.17	0.32
MMP14	melanoma_origin	primary	-0.11	0.52	0.83	0.90
MMP15	melanoma_origin	primary	-2.50	1.31	0.06	0.17
MMP16	melanoma_origin	primary	-1.27	1.34	0.34	0.51
MMP2	melanoma_origin	primary	0.44	0.24	0.07	0.17
MMP3	melanoma_origin	primary	-1.56	1.47	0.29	0.43
MMP7	melanoma_origin	primary	-1.19	0.66	0.07	0.17
MMP8	melanoma_origin	primary	-2.65	1.43	0.06	0.17
MMP9	melanoma_origin	primary	1.06	0.68	0.12	0.27
NCAM1	melanoma_origin	primary	3.05	1.13	0.01	0.11
PECAM1	melanoma_origin	primary	3.24	1.58	0.04	0.17
SELE	melanoma_origin	primary	-0.92	1.73	0.60	0.72
SELL	melanoma_origin	primary	0.21	1.25	0.87	0.91
SELP	melanoma_origin	primary	0.46	0.66	0.49	0.64
SPARC	melanoma_origin	primary	-0.33	0.39	0.40	0.56
SPG7	melanoma_origin	primary	2.77	2.18	0.20	0.36
SPP1	melanoma_origin	primary	-2.50	1.38	0.07	0.17
TGFB1	melanoma_origin	primary	0.15	0.50	0.76	0.85
THBS1	melanoma_origin	primary	0.88	1.59	0.58	0.72
THBS2	melanoma_origin	primary	-3.47	1.83	0.06	0.17
THBS3	melanoma_origin	primary	0.79	0.35	0.02	0.16
TIMP1	melanoma_origin	primary	-1.07	0.61	0.08	0.19
TIMP2	melanoma_origin	primary	0.32	0.26	0.22	0.38
TNC	melanoma_origin	primary	-2.33	1.00	0.02	0.16
VCAM1	melanoma_origin	primary	-0.10	0.49	0.83	0.90
VCAN	melanoma_origin	primary	-1.41	1.01	0.16	0.31

10. Supplementary Table S6: Results for all 86 tested genes of linear mixed-effects models fitted within each endothelial context to estimate melanoma-origin effects on expression, adjusting for adhesion-state

Gene	Stratum-type	Stratum	estimate	se	p value	q value
ADAMTS1	organ	brain	-1.77	1.76	0.31	0.59

ADAMTS13	organ	brain	-0.30	1.82	0.87	0.88
ADAMTS8	organ	brain	-0.26	0.89	0.77	0.85
CD44	organ	brain	0.18	1.22	0.89	0.88
CDH1	organ	brain	0.41	4.02	0.92	0.88
CNTN1	organ	brain	3.29	3.78	0.38	0.65
COL11A1	organ	brain	0.79	1.52	0.60	0.80
COL12A1	organ	brain	-1.42	1.05	0.17	0.46
COL14A1	organ	brain	2.36	2.53	0.35	0.63
COL15A1	organ	brain	0.42	5.06	0.93	0.88
COL16A1	organ	brain	-2.08	1.76	0.24	0.53
COL1A1	organ	brain	-1.30	1.00	0.19	0.47
COL4A2	organ	brain	-0.04	4.15	0.99	0.88
COL5A1	organ	brain	-6.47	2.97	0.03	0.13
COL6A1	organ	brain	-0.15	1.97	0.94	0.88
COL7A1	organ	brain	2.82	4.71	0.55	0.79
COL8A1	organ	brain	-4.85	1.73	0.00	0.04
CTGF	organ	brain	-1.89	0.78	0.01	0.09
CTNNA1	organ	brain	0.32	1.50	0.83	0.88
CTNNB1	organ	brain	-0.23	1.29	0.86	0.88
CTNND1	organ	brain	0.01	0.64	0.99	0.88
CTNND2	organ	brain	0.85	2.83	0.76	0.85
ECM1	organ	brain	7.41	3.87	0.06	0.21
FN1	organ	brain	-2.71	2.93	0.36	0.63
HAS1	organ	brain	0.39	0.78	0.62	0.80
ICAM1	organ	brain	0.61	1.63	0.71	0.85
ITGA1	organ	brain	-3.65	3.23	0.26	0.55
ITGA10	organ	brain	-4.46	4.08	0.27	0.56
ITGA11	organ	brain	-6.10	1.98	0.00	0.02
ITGA2	organ	brain	2.53	1.16	0.03	0.13
ITGA3	organ	brain	2.62	0.99	0.01	0.06
ITGA4	organ	brain	7.59	4.70	0.11	0.32
ITGA5	organ	brain	-0.90	0.10	0.00	0.00
ITGA6	organ	brain	1.47	0.77	0.06	0.21
ITGA7	organ	brain	2.97	1.51	0.05	0.20
ITGA8	organ	brain	3.47	3.53	0.33	0.60
ITGA9	organ	brain	-0.82	2.71	0.76	0.85
ITGAD	organ	brain	-1.54	0.85	0.07	0.24
ITGAL	organ	brain	-0.25	1.46	0.87	0.88
ITGAM	organ	brain	1.50	2.32	0.52	0.79
ITGAV	organ	brain	-1.82	2.72	0.51	0.79
ITGAX	organ	brain	-7.90	3.17	0.01	0.08
ITGB1	organ	brain	-1.70	0.72	0.02	0.10

ITGB2	organ	brain	0.14	1.87	0.94	0.88
ITGB3	organ	brain	3.91	3.25	0.23	0.53
ITGB4	organ	brain	-4.25	4.00	0.29	0.58
ITGB5	organ	brain	0.63	1.35	0.64	0.80
ITGB6	organ	brain	-1.00	2.02	0.62	0.80
ITGB7	organ	brain	2.22	4.10	0.59	0.80
ITGB8	organ	brain	6.57	1.89	0.00	0.01
LAMA1	organ	brain	6.57	4.79	0.17	0.46
LAMA2	organ	brain	-4.53	2.06	0.03	0.13
LAMA3	organ	brain	-0.09	3.19	0.98	0.88
LAMB1	organ	brain	-0.65	1.39	0.64	0.80
LAMB3	organ	brain	1.29	1.98	0.51	0.79
LAMC1	organ	brain	0.67	1.27	0.60	0.80
MMP1	organ	brain	-6.45	7.61	0.40	0.66
MMP10	organ	brain	-1.99	1.19	0.09	0.31
MMP11	organ	brain	-0.25	2.27	0.91	0.88
MMP12	organ	brain	-2.86	0.84	0.00	0.01
MMP13	organ	brain	4.53	4.02	0.26	0.55
MMP14	organ	brain	0.21	0.69	0.76	0.85
MMP15	organ	brain	0.87	3.63	0.81	0.88
MMP16	organ	brain	1.02	1.93	0.60	0.80
MMP2	organ	brain	-5.57	0.42	0.00	0.00
MMP3	organ	brain	-7.80	0.63	0.00	0.00
MMP7	organ	brain	4.31	1.22	0.00	0.01
MMP8	organ	brain	0.07	2.74	0.98	0.88
MMP9	organ	brain	-1.42	2.09	0.50	0.79
NCAM1	organ	brain	-8.84	1.38	0.00	0.00
PECAM1	organ	brain	3.76	3.07	0.22	0.52
SELE	organ	brain	2.04	2.33	0.38	0.65
SELL	organ	brain	-1.44	4.50	0.75	0.85
SELP	organ	brain	-1.30	0.96	0.18	0.46
SPARC	organ	brain	-3.28	2.04	0.11	0.32
SPG7	organ	brain	-1.33	1.02	0.19	0.47
SPP1	organ	brain	10.35	1.88	0.00	0.00
TGFB1	organ	brain	-0.53	0.85	0.53	0.79
THBS1	organ	brain	-6.63	3.09	0.03	0.13
THBS2	organ	brain	0.66	5.29	0.90	0.88
THBS3	organ	brain	-0.51	0.85	0.55	0.79
TIMP1	organ	brain	0.39	1.20	0.74	0.85
TIMP2	organ	brain	0.20	0.20	0.31	0.59
TNC	organ	brain	0.85	2.86	0.77	0.85
VCAM1	organ	brain	0.03	2.05	0.99	0.88

https://doi.org/10.3390/biomedicines14071409

VCAN	organ	brain	3.42	2.25	0.13	0.38
ADAMTS1	organ	hepatic	-1.38	1.61	0.39	0.69
ADAMTS13	organ	hepatic	-1.57	0.74	0.03	0.22
ADAMTS8	organ	hepatic	-0.85	1.53	0.58	0.86
CD44	organ	hepatic	-0.32	1.35	0.81	0.87
CDH1	organ	hepatic	1.28	4.24	0.76	0.87
CNTN1	organ	hepatic	1.13	4.09	0.78	0.87
COL11A1	organ	hepatic	0.12	1.29	0.93	0.89
COL12A1	organ	hepatic	-1.22	0.87	0.16	0.43
COL14A1	organ	hepatic	1.31	1.87	0.48	0.77
COL15A1	organ	hepatic	1.08	4.86	0.82	0.87
COL16A1	organ	hepatic	-1.33	1.68	0.43	0.74
COL1A1	organ	hepatic	-4.71	2.60	0.07	0.27
COL4A2	organ	hepatic	-0.16	2.59	0.95	0.90
COL5A1	organ	hepatic	-5.51	2.93	0.06	0.27
COL6A1	organ	hepatic	-0.19	2.12	0.93	0.89
COL7A1	organ	hepatic	-1.96	4.06	0.63	0.86
COL8A1	organ	hepatic	-4.76	2.67	0.08	0.27
CTGF	organ	hepatic	-1.97	1.01	0.05	0.27
CTNNA1	organ	hepatic	0.22	1.27	0.86	0.87
CTNNB1	organ	hepatic	-0.13	1.00	0.90	0.89
CTNND1	organ	hepatic	0.48	0.62	0.44	0.75
CTNND2	organ	hepatic	1.25	2.49	0.62	0.86
ECM1	organ	hepatic	1.93	4.97	0.70	0.86
FN1	organ	hepatic	-3.97	1.42	0.01	0.05
HAS1	organ	hepatic	-0.45	0.63	0.48	0.77
ICAM1	organ	hepatic	0.85	1.49	0.57	0.86
ITGA1	organ	hepatic	-1.32	2.77	0.63	0.86
ITGA10	organ	hepatic	0.60	2.43	0.80	0.87
ITGA11	organ	hepatic	-7.60	1.51	0.00	0.00
ITGA2	organ	hepatic	2.59	1.25	0.04	0.24
ITGA3	organ	hepatic	0.55	2.97	0.85	0.87
ITGA4	organ	hepatic	6.74	3.68	0.07	0.27
ITGA5	organ	hepatic	-0.54	0.16	0.00	0.01
ITGA6	organ	hepatic	0.92	0.71	0.20	0.47
ITGA7	organ	hepatic	3.12	1.76	0.08	0.27
ITGA8	organ	hepatic	0.80	4.35	0.85	0.87
ITGA9	organ	hepatic	-0.63	2.60	0.81	0.87
ITGAD	organ	hepatic	-0.97	0.85	0.25	0.53
ITGAL	organ	hepatic	-0.70	1.82	0.70	0.86
ITGAM	organ	hepatic	1.40	1.32	0.29	0.57
ITGAV	organ	hepatic	-3.82	2.08	0.07	0.27

https://doi.org/10.3390/biomedicines14071409

ITGAX	organ	hepatic	0.87	2.32	0.71	0.86
ITGB1	organ	hepatic	-1.41	0.66	0.03	0.22
ITGB2	organ	hepatic	-0.86	0.55	0.12	0.37
ITGB3	organ	hepatic	4.23	2.86	0.14	0.40
ITGB4	organ	hepatic	-7.06	1.87	0.00	0.00
ITGB5	organ	hepatic	-0.11	0.66	0.86	0.87
ITGB6	organ	hepatic	-0.65	1.62	0.69	0.86
ITGB7	organ	hepatic	0.56	0.52	0.28	0.57
ITGB8	organ	hepatic	5.99	1.86	0.00	0.02
LAMA1	organ	hepatic	4.70	3.97	0.24	0.51
LAMA2	organ	hepatic	7.82	4.93	0.11	0.37
LAMA3	organ	hepatic	3.45	3.47	0.32	0.59
LAMB1	organ	hepatic	-1.43	1.21	0.24	0.51
LAMB3	organ	hepatic	0.12	2.31	0.96	0.90
LAMC1	organ	hepatic	-3.91	1.39	0.00	0.05
MMP1	organ	hepatic	-4.74	5.10	0.35	0.64
MMP10	organ	hepatic	-2.97	1.06	0.01	0.05
MMP11	organ	hepatic	2.26	1.70	0.18	0.46
MMP12	organ	hepatic	-1.79	1.16	0.12	0.38
MMP13	organ	hepatic	2.86	1.48	0.05	0.27
MMP14	organ	hepatic	0.44	0.98	0.65	0.86
MMP15	organ	hepatic	0.50	2.75	0.86	0.87
MMP16	organ	hepatic	-0.62	1.43	0.67	0.86
MMP2	organ	hepatic	-4.94	0.30	0.00	0.00
MMP3	organ	hepatic	-4.33	2.13	0.04	0.24
MMP7	organ	hepatic	2.42	1.99	0.22	0.51
MMP8	organ	hepatic	-2.42	1.66	0.15	0.40
MMP9	organ	hepatic	-1.71	1.34	0.20	0.47
NCAM1	organ	hepatic	5.43	5.16	0.29	0.57
PECAM1	organ	hepatic	0.30	2.17	0.89	0.89
SELE	organ	hepatic	0.89	2.53	0.72	0.87
SELL	organ	hepatic	-1.69	4.05	0.68	0.86
SELP	organ	hepatic	-1.83	1.10	0.10	0.33
SPARC	organ	hepatic	-1.44	2.00	0.47	0.77
SPG7	organ	hepatic	2.91	2.91	0.32	0.59
SPP1	organ	hepatic	9.74	1.44	0.00	0.00
TGFB1	organ	hepatic	0.00	0.42	1.00	0.93
THBS1	organ	hepatic	-7.04	3.08	0.02	0.18
THBS2	organ	hepatic	0.90	5.17	0.86	0.87
THBS3	organ	hepatic	0.32	0.72	0.66	0.86
TIMP1	organ	hepatic	0.55	1.08	0.61	0.86
TIMP2	organ	hepatic	0.45	0.31	0.14	0.40

TNC	organ	hepatic	1.35	2.61	0.60	0.86
VCAM1	organ	hepatic	-1.84	3.81	0.63	0.86
VCAN	organ	hepatic	2.86	2.11	0.18	0.46
ADAMTS1	organ	pulmonary	-1.27	1.73	0.46	0.89
ADAMTS13	organ	pulmonary	-1.75	0.98	0.07	0.57
ADAMTS8	organ	pulmonary	-1.04	1.03	0.31	0.86
CD44	organ	pulmonary	0.66	0.70	0.34	0.86
CDH1	organ	pulmonary	1.87	4.16	0.65	0.90
CNTN1	organ	pulmonary	1.75	3.93	0.66	0.90
COL11A1	organ	pulmonary	0.42	0.74	0.57	0.89
COL12A1	organ	pulmonary	-1.12	0.45	0.01	0.15
COL14A1	organ	pulmonary	1.52	1.93	0.43	0.89
COL15A1	organ	pulmonary	-1.79	4.26	0.67	0.90
COL16A1	organ	pulmonary	-1.07	1.14	0.35	0.86
COL1A1	organ	pulmonary	-1.07	7.74	0.89	0.95
COL4A2	organ	pulmonary	-0.19	3.70	0.96	0.95
COL5A1	organ	pulmonary	-2.24	0.75	0.00	0.06
COL6A1	organ	pulmonary	-0.73	1.16	0.53	0.89
COL7A1	organ	pulmonary	5.19	4.57	0.26	0.83
COL8A1	organ	pulmonary	-2.15	0.92	0.02	0.21
CTGF	organ	pulmonary	-0.66	0.52	0.21	0.83
CTNNA1	organ	pulmonary	0.35	1.04	0.74	0.91
CTNNB1	organ	pulmonary	0.08	0.88	0.93	0.95
CTNND1	organ	pulmonary	0.52	0.42	0.21	0.83
CTNND2	organ	pulmonary	1.97	2.85	0.49	0.89
ECM1	organ	pulmonary	0.63	2.28	0.78	0.94
FN1	organ	pulmonary	-0.93	2.64	0.72	0.91
HAS1	organ	pulmonary	-0.52	1.06	0.62	0.90
ICAM1	organ	pulmonary	-0.88	1.47	0.55	0.89
ITGA1	organ	pulmonary	-2.45	1.64	0.14	0.71
ITGA10	organ	pulmonary	-1.33	2.78	0.63	0.90
ITGA11	organ	pulmonary	-5.63	1.86	0.00	0.06
ITGA2	organ	pulmonary	1.06	0.63	0.10	0.61
ITGA3	organ	pulmonary	0.53	1.65	0.75	0.91
ITGA4	organ	pulmonary	3.59	3.04	0.24	0.83
ITGA5	organ	pulmonary	-0.50	0.24	0.04	0.35
ITGA6	organ	pulmonary	-1.84	3.03	0.54	0.89
ITGA7	organ	pulmonary	1.64	2.29	0.47	0.89
ITGA8	organ	pulmonary	2.28	3.82	0.55	0.89
ITGA9	organ	pulmonary	-1.94	2.95	0.51	0.89
ITGAD	organ	pulmonary	-0.08	0.87	0.93	0.95
ITGAL	organ	pulmonary	-0.50	0.54	0.35	0.86

https://doi.org/10.3390/biomedicines14071409

ITGAM	organ	pulmonary	1.73	1.37	0.21	0.83
ITGAV	organ	pulmonary	0.11	2.34	0.96	0.95
ITGAX	organ	pulmonary	-0.16	3.97	0.97	0.95
ITGB1	organ	pulmonary	-0.96	0.35	0.01	0.09
ITGB2	organ	pulmonary	0.53	0.58	0.36	0.86
ITGB3	organ	pulmonary	-1.15	2.52	0.65	0.90
ITGB4	organ	pulmonary	-2.54	4.48	0.57	0.89
ITGB5	organ	pulmonary	0.68	0.96	0.48	0.89
ITGB6	organ	pulmonary	0.97	1.28	0.45	0.89
ITGB7	organ	pulmonary	-0.76	1.02	0.46	0.89
ITGB8	organ	pulmonary	4.05	2.47	0.10	0.61
LAMA1	organ	pulmonary	8.03	2.81	0.00	0.07
LAMA2	organ	pulmonary	-0.69	4.03	0.86	0.95
LAMA3	organ	pulmonary	-5.23	4.54	0.25	0.83
LAMB1	organ	pulmonary	-0.24	2.27	0.92	0.95
LAMB3	organ	pulmonary	0.25	1.88	0.89	0.95
LAMC1	organ	pulmonary	2.38	2.26	0.29	0.86
MMP1	organ	pulmonary	-3.41	3.72	0.36	0.86
MMP10	organ	pulmonary	-1.67	1.23	0.17	0.83
MMP11	organ	pulmonary	-2.52	2.01	0.21	0.83
MMP12	organ	pulmonary	-1.70	0.90	0.06	0.49
MMP13	organ	pulmonary	-1.65	2.47	0.50	0.89
MMP14	organ	pulmonary	-0.06	0.45	0.90	0.95
MMP15	organ	pulmonary	-0.88	3.58	0.81	0.94
MMP16	organ	pulmonary	1.75	1.13	0.12	0.68
MMP2	organ	pulmonary	-2.68	0.67	0.00	0.01
MMP3	organ	pulmonary	-2.42	2.04	0.24	0.83
MMP7	organ	pulmonary	3.61	2.12	0.09	0.61
MMP8	organ	pulmonary	-2.01	4.55	0.66	0.90
MMP9	organ	pulmonary	-1.15	1.66	0.49	0.89
NCAM1	organ	pulmonary	3.65	6.80	0.59	0.90
PECAM1	organ	pulmonary	-2.38	2.44	0.33	0.86
SELE	organ	pulmonary	2.18	6.88	0.75	0.91
SELL	organ	pulmonary	-0.60	4.80	0.90	0.95
SELP	organ	pulmonary	-0.37	1.10	0.74	0.91
SPARC	organ	pulmonary	-1.41	1.97	0.47	0.89
SPG7	organ	pulmonary	-3.87	3.56	0.28	0.86
SPP1	organ	pulmonary	9.14	2.71	0.00	0.03
TGFB1	organ	pulmonary	0.23	0.90	0.80	0.94
THBS1	organ	pulmonary	-2.85	2.51	0.25	0.83
THBS2	organ	pulmonary	-1.37	3.68	0.71	0.91
THBS3	organ	pulmonary	-0.06	0.63	0.92	0.95

TIMP1	organ	pulmonary	-0.52	1.23	0.67	0.90
TIMP2	organ	pulmonary	0.40	0.63	0.53	0.89
TNC	organ	pulmonary	0.27	2.14	0.90	0.95
VCAM1	organ	pulmonary	-1.29	2.26	0.57	0.89
VCAN	organ	pulmonary	0.14	1.70	0.93	0.95
ADAMTS1	adhesion	adherent	-2.17	1.89	0.25	0.59
ADAMTS13	adhesion	adherent	-1.46	1.37	0.29	0.59
ADAMTS8	adhesion	adherent	-0.70	0.73	0.34	0.62
CD44	adhesion	adherent	0.73	1.18	0.54	0.73
CDH1	adhesion	adherent	2.38	4.72	0.61	0.74
CNTN1	adhesion	adherent	4.21	4.95	0.39	0.67
COL11A1	adhesion	adherent	1.34	1.28	0.30	0.60
COL12A1	adhesion	adherent	-1.46	1.24	0.24	0.59
COL14A1	adhesion	adherent	2.70	2.47	0.28	0.59
COL15A1	adhesion	adherent	2.89	4.84	0.55	0.73
COL16A1	adhesion	adherent	-0.89	2.01	0.66	0.75
COL1A1	adhesion	adherent	-0.46	2.55	0.86	0.87
COL4A2	adhesion	adherent	1.75	3.81	0.65	0.74
COL5A1	adhesion	adherent	-6.03	2.36	0.01	0.08
COL6A1	adhesion	adherent	-0.96	1.92	0.62	0.74
COL7A1	adhesion	adherent	3.77	3.92	0.34	0.62
COL8A1	adhesion	adherent	-2.63	1.37	0.05	0.23
CTGF	adhesion	adherent	-1.14	0.49	0.02	0.12
CTNNA1	adhesion	adherent	1.18	1.51	0.43	0.67
CTNNB1	adhesion	adherent	0.70	1.31	0.59	0.74
CTNND1	adhesion	adherent	0.70	0.77	0.36	0.63
CTNND2	adhesion	adherent	2.11	2.70	0.43	0.67
ECM1	adhesion	adherent	4.53	2.97	0.13	0.44
FN1	adhesion	adherent	-2.02	2.89	0.48	0.71
HAS1	adhesion	adherent	0.15	0.48	0.74	0.80
ICAM1	adhesion	adherent	1.31	1.72	0.45	0.68
ITGA1	adhesion	adherent	-1.43	2.88	0.62	0.74
ITGA10	adhesion	adherent	-3.58	2.71	0.19	0.52
ITGA11	adhesion	adherent	-5.68	2.43	0.02	0.12
ITGA2	adhesion	adherent	1.99	0.91	0.03	0.15
ITGA3	adhesion	adherent	2.18	1.90	0.25	0.59
ITGA4	adhesion	adherent	9.22	2.70	0.00	0.01
ITGA5	adhesion	adherent	-0.56	0.16	0.00	0.00
ITGA6	adhesion	adherent	1.76	0.37	0.00	0.00
ITGA7	adhesion	adherent	3.06	2.32	0.19	0.52
ITGA8	adhesion	adherent	3.74	3.95	0.34	0.62
ITGA9	adhesion	adherent	0.13	2.45	0.96	0.89

https://doi.org/10.3390/biomedicines14071409

ITGAD	adhesion	adherent	-0.36	1.12	0.75	0.80
ITGAL	adhesion	adherent	-0.28	1.31	0.83	0.87
ITGAM	adhesion	adherent	2.11	2.21	0.34	0.62
ITGAV	adhesion	adherent	-0.43	2.74	0.88	0.87
ITGAX	adhesion	adherent	-3.08	2.60	0.24	0.59
ITGB1	adhesion	adherent	-1.81	0.35	0.00	0.00
ITGB2	adhesion	adherent	0.15	1.42	0.92	0.88
ITGB3	adhesion	adherent	4.68	2.29	0.04	0.19
ITGB4	adhesion	adherent	-2.78	1.98	0.16	0.48
ITGB5	adhesion	adherent	0.57	1.16	0.62	0.74
ITGB6	adhesion	adherent	0.19	1.42	0.90	0.87
ITGB7	adhesion	adherent	2.03	1.39	0.15	0.47
ITGB8	adhesion	adherent	6.74	1.20	0.00	0.00
LAMA1	adhesion	adherent	8.53	3.50	0.01	0.10
LAMA2	adhesion	adherent	-0.16	2.37	0.95	0.89
LAMA3	adhesion	adherent	2.05	3.34	0.54	0.73
LAMB1	adhesion	adherent	-0.06	2.08	0.98	0.89
LAMB3	adhesion	adherent	1.87	2.27	0.41	0.67
LAMC1	adhesion	adherent	1.07	1.34	0.42	0.67
MMP1	adhesion	adherent	-7.05	6.26	0.26	0.59
MMP10	adhesion	adherent	-2.17	1.32	0.10	0.38
MMP11	adhesion	adherent	2.50	1.56	0.11	0.40
MMP12	adhesion	adherent	-1.84	0.61	0.00	0.02
MMP13	adhesion	adherent	2.11	2.19	0.34	0.62
MMP14	adhesion	adherent	0.01	0.43	0.98	0.89
MMP15	adhesion	adherent	2.28	3.42	0.51	0.71
MMP16	adhesion	adherent	0.83	0.76	0.28	0.59
MMP2	adhesion	adherent	-4.83	0.50	0.00	0.00
MMP3	adhesion	adherent	-4.24	1.11	0.00	0.00
MMP7	adhesion	adherent	3.36	2.26	0.14	0.46
MMP8	adhesion	adherent	1.34	3.76	0.72	0.79
MMP9	adhesion	adherent	-1.07	2.30	0.64	0.74
NCAM1	adhesion	adherent	-0.84	4.24	0.84	0.87
PECAM1	adhesion	adherent	-1.31	1.95	0.50	0.71
SELE	adhesion	adherent	0.91	1.60	0.57	0.74
SELL	adhesion	adherent	0.04	5.06	0.99	0.89
SELP	adhesion	adherent	-1.33	0.77	0.08	0.34
SPARC	adhesion	adherent	-1.09	2.57	0.67	0.75
SPG7	adhesion	adherent	-1.40	2.71	0.61	0.74
SPP1	adhesion	adherent	11.22	1.50	0.00	0.00
TGFB1	adhesion	adherent	-0.08	0.83	0.92	0.88
THBS1	adhesion	adherent	-6.53	3.01	0.03	0.15

THBS2	adhesion	adherent	0.77	4.49	0.86	0.87
THBS3	adhesion	adherent	-0.50	0.36	0.16	0.48
TIMP1	adhesion	adherent	0.89	1.11	0.42	0.67
TIMP2	adhesion	adherent	0.27	0.40	0.51	0.71
TNC	adhesion	adherent	2.73	2.45	0.26	0.59
VCAM1	adhesion	adherent	-0.52	3.49	0.88	0.87
VCAN	adhesion	adherent	3.28	1.61	0.04	0.19
ADAMTS1	adhesion	non_adherent	-1.66	2.01	0.41	0.92
ADAMTS13	adhesion	non_adherent	-0.62	1.17	0.60	0.92
ADAMTS8	adhesion	non_adherent	-0.42	1.23	0.73	0.92
CD44	adhesion	non_adherent	0.03	1.35	0.98	0.92
CDH1	adhesion	non_adherent	3.15	4.84	0.52	0.92
CNTN1	adhesion	non_adherent	2.72	4.14	0.51	0.92
COL11A1	adhesion	non_adherent	0.13	1.29	0.92	0.92
COL12A1	adhesion	non_adherent	-1.44	0.37	0.00	0.00
COL14A1	adhesion	non_adherent	2.28	2.48	0.36	0.92
COL15A1	adhesion	non_adherent	-0.86	6.49	0.89	0.92
COL16A1	adhesion	non_adherent	-1.00	1.43	0.49	0.92
COL1A1	adhesion	non_adherent	-2.67	4.59	0.56	0.92
COL4A2	adhesion	non_adherent	0.09	4.19	0.98	0.92
COL5A1	adhesion	non_adherent	-4.82	2.45	0.05	0.32
COL6A1	adhesion	non_adherent	-0.91	2.14	0.67	0.92
COL7A1	adhesion	non_adherent	2.61	6.20	0.67	0.92
COL8A1	adhesion	non_adherent	-5.62	1.86	0.00	0.03
CTGF	adhesion	non_adherent	-1.90	0.80	0.02	0.16
CTNNA1	adhesion	non_adherent	0.19	1.45	0.90	0.92
CTNNB1	adhesion	non_adherent	-0.26	1.12	0.82	0.92
CTNND1	adhesion	non_adherent	0.13	0.48	0.78	0.92
CTNND2	adhesion	non_adherent	2.37	3.11	0.45	0.92
ECM1	adhesion	non_adherent	3.29	5.57	0.55	0.92
FN1	adhesion	non_adherent	-1.85	2.42	0.45	0.92
HAS1	adhesion	non_adherent	-0.37	0.93	0.69	0.92
ICAM1	adhesion	non_adherent	-0.55	2.00	0.78	0.92
ITGA1	adhesion	non_adherent	-2.02	2.60	0.44	0.92
ITGA10	adhesion	non_adherent	-0.10	2.37	0.97	0.92
ITGA11	adhesion	non_adherent	-6.37	1.27	0.00	0.00
ITGA2	adhesion	non_adherent	1.48	1.27	0.24	0.80
ITGA3	adhesion	non_adherent	0.42	1.87	0.82	0.92
ITGA4	adhesion	non_adherent	3.43	5.50	0.53	0.92
ITGA5	adhesion	non_adherent	-0.71	0.12	0.00	0.00
ITGA6	adhesion	non_adherent	-0.95	1.57	0.55	0.92
ITGA7	adhesion	non_adherent	3.36	1.94	0.08	0.50

https://doi.org/10.3390/biomedicines14071409

ITGA8	adhesion	non_adherent	3.18	4.67	0.50	0.92
ITGA9	adhesion	non_adherent	-1.47	4.24	0.73	0.92
ITGAD	adhesion	non_adherent	-1.23	0.86	0.15	0.75
ITGAL	adhesion	non_adherent	-0.01	1.39	0.99	0.92
ITGAM	adhesion	non_adherent	1.85	0.80	0.02	0.16
ITGAV	adhesion	non_adherent	-1.69	2.55	0.51	0.92
ITGAX	adhesion	non_adherent	-2.38	1.93	0.22	0.80
ITGB1	adhesion	non_adherent	-1.02	0.91	0.26	0.82
ITGB2	adhesion	non_adherent	-0.17	0.70	0.81	0.92
ITGB3	adhesion	non_adherent	0.52	4.44	0.91	0.92
ITGB4	adhesion	non_adherent	-4.86	5.25	0.35	0.92
ITGB5	adhesion	non_adherent	0.59	0.95	0.53	0.92
ITGB6	adhesion	non_adherent	0.19	2.26	0.93	0.92
ITGB7	adhesion	non_adherent	-0.93	1.05	0.37	0.92
ITGB8	adhesion	non_adherent	4.38	3.50	0.21	0.80
LAMA1	adhesion	non_adherent	6.88	4.90	0.16	0.75
LAMA2	adhesion	non_adherent	0.78	2.95	0.79	0.92
LAMA3	adhesion	non_adherent	-2.17	4.12	0.60	0.92
LAMB1	adhesion	non_adherent	-0.55	1.46	0.71	0.92
LAMB3	adhesion	non_adherent	0.33	2.43	0.89	0.92
LAMC1	adhesion	non_adherent	-1.33	1.54	0.39	0.92
MMP1	adhesion	non_adherent	-6.50	6.32	0.30	0.92
MMP10	adhesion	non_adherent	-2.73	0.76	0.00	0.00
MMP11	adhesion	non_adherent	-2.91	1.29	0.02	0.17
MMP12	adhesion	non_adherent	-2.39	1.77	0.18	0.77
MMP13	adhesion	non_adherent	1.50	1.14	0.19	0.77
MMP14	adhesion	non_adherent	0.19	0.55	0.72	0.92
MMP15	adhesion	non_adherent	-0.33	4.26	0.94	0.92
MMP16	adhesion	non_adherent	0.64	2.02	0.75	0.92
MMP2	adhesion	non_adherent	-3.97	0.59	0.00	0.00
MMP3	adhesion	non_adherent	-5.38	2.31	0.02	0.16
MMP7	adhesion	non_adherent	2.46	1.53	0.11	0.60
MMP8	adhesion	non_adherent	-3.42	2.86	0.23	0.80
MMP9	adhesion	non_adherent	-1.00	1.78	0.57	0.92
NCAM1	adhesion	non_adherent	2.63	5.20	0.61	0.92
PECAM1	adhesion	non_adherent	1.84	1.58	0.24	0.80
SELE	adhesion	non_adherent	1.09	4.32	0.80	0.92
SELL	adhesion	non_adherent	0.48	5.22	0.93	0.92
SELP	adhesion	non_adherent	-1.21	1.61	0.45	0.92
SPARC	adhesion	non_adherent	-1.63	1.99	0.41	0.92
SPG7	adhesion	non_adherent	-0.20	0.52	0.70	0.92
SPP1	adhesion	non_adherent	8.85	2.60	0.00	0.01

TGFB1	adhesion	non_adherent	-0.06	0.32	0.85	0.92
THBS1	adhesion	non_adherent	-5.93	3.76	0.11	0.60
THBS2	adhesion	non_adherent	-2.79	6.51	0.67	0.92
THBS3	adhesion	non_adherent	0.10	1.12	0.93	0.92
TIMP1	adhesion	non_adherent	-0.26	1.68	0.88	0.92
TIMP2	adhesion	non_adherent	0.46	0.48	0.34	0.92
TNC	adhesion	non_adherent	-0.01	3.44	1.00	0.92
VCAM1	adhesion	non_adherent	0.34	2.52	0.89	0.92
VCAN	adhesion	non_adherent	1.65	3.16	0.60	0.92

https://doi.org/10.3390/biomedicines14071409
